# Woodylides A–C, New Cytotoxic Linear Polyketides from the South China Sea Sponge *Plakortis simplex*

**DOI:** 10.3390/md10051027

**Published:** 2012-05-07

**Authors:** Hao-Bing Yu, Xiang-Fang Liu, Ying Xu, Jian-Hong Gan, Wei-Hua Jiao, Yang Shen, Hou-Wen Lin

**Affiliations:** 1 Laboratory of Marine Drugs, Department of Pharmacy, Changzheng Hospital, Second Military Medical University, Shanghai 200003, China; Email: yuhaobing1986@126.com (H.-B.Y.); xy30490@126.com (Y.X.); jhgan@shou.edu.cn (J.-H.G.); weihuajiao@hotmail.com (W.-H.J.); 2 Department of Pharmacy, Shanghai Children’s Hospital, Shanghai Jiao Tong University, Shanghai 200040, China; Email: liuxiangfang0107@126.com; 3 College of Food Science & Technology, Shanghai Ocean University, Shanghai 201306, China

**Keywords:** *Plakortis simplex*, woodylides, cytotoxicity, PTP1B inhibitory activity

## Abstract

Three new polyketides, woodylides A–C (**1–3**), were isolated from the ethanol extract of the South China Sea sponge *Plakortis simplex*. The structures were elucidated by spectroscopic data (IR, 1D and 2D NMR, and HRESIMS). The absolute configurations at C-3 of **1** and **3** were determined by the modified Mosher’s method. Antifungal, cytotoxic, and PTP1B inhibitory activities of these polyketides were evaluated. Compounds **1** and **3** showed antifungal activity against fungi *Cryptococcus neoformans* with IC_50_ values of 3.67 and 10.85 µg/mL, respectively. In the cytotoxicity test, compound **1** exhibited a moderate effect against the HeLa cell line with an IC_50_ value of 11.2 µg/mL, and compound **3** showed cytotoxic activity against the HCT-116 human colon tumor cell line and PTP1B inhibitory activity with IC_50_ values of 9.4 and 4.7 µg/mL, respectively.

## 1. Introduction

**Figure 1:**
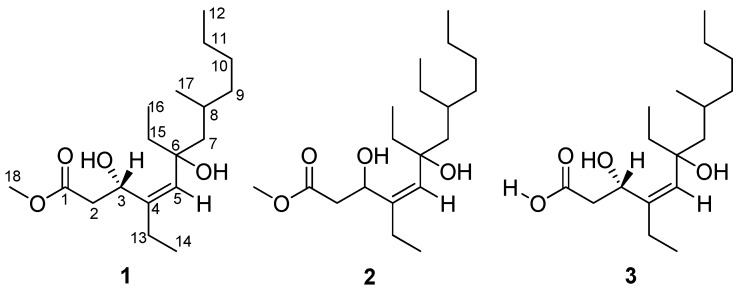
Structures of woodylides A (**1**), B (**2**) and C (**3**).

Polyketides are a structurally diverse family of natural products with various biological activities and pharmacological properties, biogenetically derived from acetate, propionate and butyrate units [1,2,3]. Marine sponges provide a wide range of polyketides with antibacterial [4], antiviral [5], antitumor [6], antimalarial [7], and taxol-like microtubule-stabilizing activities [8]. The sponge-derived polyketides often contain cyclic peroxides and lactone functionalities, linear and bicyclic carbon frameworks [1,9], and macrolide and aromatic groups in some cases [8,10]. A prolific source of new and bioactive polyketides derived from sponges of the genus *Plakortis* attracted our attention. As part of our ongoing search for new pharmacologically active lead compounds from the marine sponges collected off Xisha Islands in the South China Sea [11,12], we investigated polyketides from the marine sponge *Plakortis simplex*. A preliminary study led to the isolation of two new polyketides named simplextones A and B with an unusual cyclopentane skeleton [6]. The interesting chemical and bioactive significance of *P. simplex* prompted us to continue the study of this sponge, which has led to the isolation of three new linear polyketides, named as woodylide A (**1**), B (**2**) and C (**3**) (Figure 1) [13], which are the acyclic diol analogues of the cyclic polyketide peroxides isolated from the genus of *Plakortis* [14,15]. This article describes the isolation, identification and bioactivity of the new compounds.

## 2. Results and Discussion

**Figure 2:**
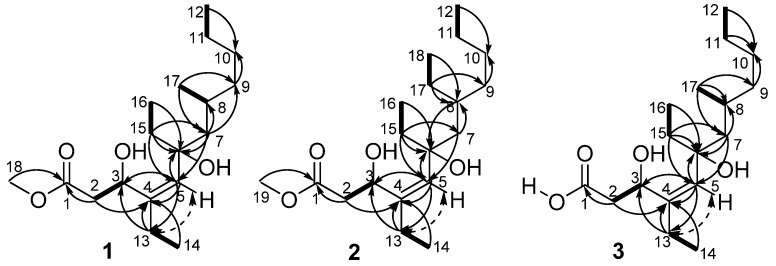
COSY (▬), Key HMBC (→), and selected NOE (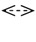) correlations of **1**, **2**, and **3**.

Compound **1** was obtained as a colorless oil. The positive HRESIMS exhibited a pseudomolecular ion peak at *m/z* 337.2354, [M + Na]^+^ (calcd 337.2355 [16]), consistent with a molecular formula of C_18_H_34_O_4_, indicating two double bond equivalents. The IR absorption bands supported the existence of hydroxyl (3275 cm^−1^), carbonyl (1742 cm^−1^), and olefinic (1650 cm^−1^) functional groups. The ^13^C NMR and DEPT spectra indicated the presence of 18 carbon atoms, corresponding to a total of one carbonyl (δ_C_ 173.6), one olefinic quaternary carbon (δ_C_ 140.5), one olefinic methine (δ_C_ 132.4), one oxygenated quaternary carbon (δ_C_ 77.0), one oxymethine (δ_C_ 68.8), one methoxyl (δ_C_ 51.8), one aliphatic methine (δ_C_ 29.2), seven aliphatic methylenes (δ_C_ 23.0, 29.4, 29.4, 36.5, 38.5, 40.7, and 48.9), and four methyl carbons (δ_C_ 8.3, 13.7, 14.2, and 22.2) (Table 1). The ^1^H NMR spectrum displayed resonances for one methyl group attached to a tertiary carbon at δ_H_ 0.98 (3H, d, *J* = 6.5 Hz), three methyl groups attached to secondary carbons at δ_H_ 0.88 (3H, t, *J* = 7.0 Hz), δ_H_ 0.88 (3H, t, *J* = 7.0 Hz overlapped), and δ_H_ 1.05 (3H, t, *J* = 7.0 Hz), one methoxyl group at δ_H_ 3.71 (3H, s), and one olefinic proton at δ_H_ 5.11 (1H, s). The two double bond equivalents of **1** were accounted for one double bond and one carbonyl group, revealing the linearity of its carbon scaffold. Analysis of the COSY and HSQC spectra revealed the presence of five spin systems in the structure: H_2_-2/H-3, H-8/H_3_-17, H_2_-11/H_3_-12, H_2_-13/H_3_-14, and H_2_-15/H_3_-16 (Figure 2). The HMBC correlation from H_3_-18 (δ_H_ 3.71) to C-1 (δ_C_ 173.6) positioned the methoxyl group at C-1. The olefinic proton H-5 (δ_H_ 5.11) afforded HMBC correlations to C-3 (δ_C_ 68.8), C-4 (δ_C_ 140.5), and C-6 (δ_C_ 77.0), whereas H-7a (δ_H_ 1.34) showed HMBC correlations to C-5 (δ_C_ 132.4) and C-6, which established the connectivity of the partial structure C-3 to C-7 (δ_C_ 48.9). Obviously, the double bond was located between C-4 and C-5 on the linear carbon scaffold based on the carbon resonances of C-4 and C-5. Accordingly, the methyl acetate group was tethered to C-4 via C-3 by HMBC correlations from H-2b (δ_H_ 2.92) to C-1 and C-4, from H_2_-13 (δ_H_ 2.02) to C-3, and from H-5 to C-3. The HMBC correlations from H_3_-14 (δ_H_ 1.05) to C-4, and from H_3_-16 (δ_H_ 0.88) to C-6, unambiguously assigned the ethyl groups to C-4 and C-6, respectively. Moreover, the HMBC correlations from H_3_-17 (δ_H_ 0.98) to C-7 and C-9 (δ_C_ 38.5), and from H-7b (δ_H_ 1.55) to C-9 demonstrated the linkage of C-7, C-9 and C-17 (δ_C_ 22.2) via C-8. Even though no COSY correlation was observed between H_2_-10 (δ_H_ 1.25) and H_2_-11 (δ_H_ 1.25), the connectivity of the partial structure C-9 to C-12 (δ_C_ 14.2) was secured by the HMBC correlations of H-9b (δ_H_ 1.30)/C-10 (δ_C_ 29.4), and H_3_-12 (δ_H_ 0.88)/C-10, and by comparison of the NMR date with the known derivatives [17]. With this assignment secured, the final methine (C-3) and the oxygenated quaternary carbon (C-6) had to be substituted with hydroxyl groups to satisfy the molecular formula and shifts.

**Table 1:** NMR data for woodylides A-C (**1–3**) in CDCl_3_.

Position	1 ^a^		2 ^b^		3 ^b^	
	δ_C_	δ_H_, mult. (*J* in Hz)	δ_C_	δ_H_, mult. (*J* in Hz)	δ_C_	δ_H_, mult. (*J* in Hz)
1	173.6 qC		173.6 qC		176.4 qC	
2a	40.7 CH_2_	2.57, dd (16.5, 3.0)	40.6 CH_2_	2.57, dd (16.8, 3.0)	41.0 CH_2_	2.53, d (15.6)
2b		2.92, dd (16.5, 10.0)		2.99, dd (16.8, 10.2)		2.90, dd (15.6, 4.8)
3	68.8 CH	4.83, dd (10.0, 3.0)	68.8 CH	4.81, dd (10.2, 3.0)	68.9 CH	4.67,d (9.6)
4	140.5 qC		140.4 qC		140.9 qC	
5	132.4 CH	5.11, s	132.5 CH	5.11, s	131.3 CH	5.05, s
6	77.0 qC		77.0 qC		77.8 qC	
7a	48.9 CH_2_	1.34, dd (14.0, 7.0)	45.8 CH_2_	1.44, m	49.0 CH_2_	1.35, dd (13.8, 6.6)
7b		1.55, dd (15.0, 7.0)		1.47, m		1.56, dd (12.0, 4.8)
8	29.2 CH	1.61, m	35.0 CH	1.55, m	29.1 CH	1.60, m
9a	38.5 CH_2_	1.14, m	34.3 CH_2_	1.26, m	38.5 CH_2_	1.13, m
9b		1.30, m		1.33, m		1.30, m
10	29.4 CH_2_	1.25, m	29.0 CH_2_	1.22, m	29.3 CH_2_	1.25, m
11	23.0 CH_2_	1.25, m	23.2 CH_2_	1.26, m	23.0 CH_2_	1.25,m
12	14.2 CH_3_	0.88, t (7.0)	14.2 CH_3_	0.88, t (7.2)	14.1 CH_3_	0.86, t (7.2)
13	29.4 CH_2_	2.02, m	29.6 CH_2_	2.04, m	29.8 CH_2_	2.02, m
14	13.7 CH_3_	1.05, t (7.0)	13.6 CH_3_	1.05, t (7.8)	13.5 CH_3_	1.04, t (7.2)
15	36.5 CH_2_	1.55, m	36.6 CH_2_	1.55, m	36.5 CH_2_	1.56, m
16	8.3 CH_3_	0.88, t (7.0)	8.4 CH_3_	0.88, t (7.2)	8.3 CH_3_	0.86, t (7.2)
17	22.2 CH_3_	0.98, d (6.5)	27.7 CH_2_	1.45, m	22.1 CH_3_	0.96, d (6.6)
18	51.8 -OCH_3_	3.71, s	10.7 CH_3_	0.85, t (7.2)		
19			51.8-OCH_3_	3.71, s		

^a^ Measured at 500 MHz (^1^H) and 125 MHz (^13^C); ^b^ Measured at 600 MHz (^1^H) and 150 MHz (^13^C).

**Figure 3:**
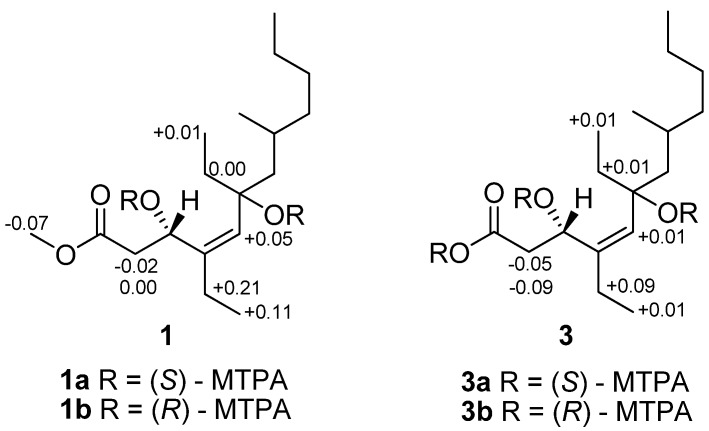
∆δ*_S_*_−*R*_ values for the MTPA derivatives of **1** and **3** in CDCl_3_.

The configuration of double bond in **1** was established on the basis of NOESY data. The *Z*-geometry of the Δ^4,5^ double bond was deduced from a NOESY correlation between H-5 and H_2_-13, as well as derived from devoid of NOESY correlation between H-5 and H-3 (δ_H_ 4.83) (Figure 2). The absolute configuration of C-3 was determined by applying the modified Mosher’s method to the secondary hydroxyl group [18]. The (*S*)- and (*R*)-MTPA esters of **1** were prepared by reaction with (*R*)- and (*S*)-MTPA chlorides, respectively. The Δδ*_S_*_−*R*_ values observed for the protons near the secondary C-3 hydroxyl group for the esters indicated the *S*-configuration for the secondary alcohol stereogenic center in **1** (Figure 3).

Compound **2** was also isolated as a colorless oil, with a molecular formula of C_19_H_36_O_4_ as determined by HRESIMS (*m/z* 351.2513, [M + Na]^+^, calcd 351.2511). Comparison of the ^1^H NMR data of **2** with those of **1**, the obvious differences were the presence of an additional methyl triplet (δ_H_ 0.85, t, *J* = 7.2 Hz) and a methylene multiplet (δ_H_ 1.45, m), as well as the absence of the methyl doublet (δ_H_ 0.98, d, *J* = 6.5 Hz), indicating an overall structure similar to **1** except for an ethyl group C-17 (δ_C_ 27.7)/C-18 (δ_C_ 10.7) attached to C-8 (δ_C_ 35.0) in **2** (Table 1). This was also supported by the HMBC correlations from both H_3_-18 (δ_H_ 0.85) and H_2_-17 (δ_H_ 1.45) to C-8, and ^1^H–^1^H COSY correlations between H_3_-18 and H_2_-17. The geometry of the trisubstituted double bond was assigned as *Z* based on the NOESY correlation between H_2_-13 (δ_H_ 2.04, m) and H-5 (δ_H_ 5.11, m) (Figure 2).

Compound **3** was assigned a molecular formula of C_17_H_32_O_4_, implying two double bond equivalents, as deduced from the HRESIMS (*m/z* 323.2200, [M + Na]^+^, calcd 323.2198) and NMR data. The ^13^C NMR and DEPT spectra exhibited 17 carbon resonances corresponding to four methyl, seven methylene, three methine, and three quaternary carbons (Table 1). The overall appearance of the NMR spectrum showed close structural similarity between **3** and **1**, except for the absence of a methoxyl resonance in **3** instead of H_3_-18 (δ_H_ 3.71)/C-18 (δ_C_ 51.8) in **1**, indicating **3** was a free carboxylic acid. This was also confirmed by the observation of a Δδ ~3 downfield shift of the C-1 from δ_C_ 173.6 to δ_C_ 176.4. The NOESY correlations observed between H_2_-13 (δ_H_ 2.02) and H-5 (δ_H_ 5.05), confirmed the *Z* geometry of the double bond at C-4 (δ_C_ 140.9)/C-5 (δ_C_ 131.3). The absolute configuration of C-3 was determined by the modified Mosher’s method [18]. Analysis of the Δδ*_S_*_−*R*_ values (Figure 3) according to Mosher’s model pointed to an *S*-configuration for C-3 in **3**.

To confirm if compound **1** could be an artifact formed from **3** during the isolation processes, a solution of **3** was kept at room temperature for three days in the presence of Si-60 or RP-18 gel in MeOH, respectively. The formation of **1** was not observed, thus suggesting that compound **1** may be a natural product and not an artifact.

The three new polyketides **1–3** were evaluated for antifungal activity against *Cryptococcus neoformans* (ATCC 90113), *Candida albicans* (Y0109), *Trichophyton rubrum* (Cmccftla) and *Microsporum gypseum* (Cmccfmza) (Table 2), for *in vitro* cytotoxic activity against human cancer cell lines, HCT-116 (colon cancer), A549 (lung carcinoma), HeLa (cervical cancer), QGY-7703 (hepatocarcinoma), and MDA231 (breast adenocarcinoma) (Table 3), and protein tyrosine phosphatase 1B (PTP1B) inhibitory activity (Table 3). Compounds **1** and **3** showed moderate antifungal activity against the fungus *C. neoformans* with IC_50_ values of 3.67 and 10.85 µg/mL, respectively, while compound **2**, bearing an ethyl group at C-8, was inactive even tested at a higher concentration. Compounds **1** and **3** showed weaker antifungal activity towards all the remaining assayed indicators. Furthermore, compound **1** showed moderate cytotoxicity (IC_50_, 11.22 µg/mL) against HeLa cell line, and compound **3** exhibited cytotoxic activity (IC_50_, 9.4 µg/mL) against HCT-116 cell line. Cytotoxicity of compound **3** against A549, HeLa, QGY-7703, and MDA231 cell lines was weaker when compared to that of **1**. In addition, compound **3** was tested for PTP1B inhibitory activity *in vitro*, with an IC_50_ value of 4.7 µg/mL. The PTP1B inhibitors are recognized as potential therapeutic agents for the treatment of type II diabetes and obesity [19].

**Table 2:** Antifungal activity of woodylides A–C (**1–3**).

Compound	Antifungal Activity			
	*C. neoformans* ^a^	*C. albicans* ^b^	*T. rubrum* ^b^	*M. gypseum* ^b^
**1**	3.67	32	32	32
**2**	NA	NT	NT	NT
**3**	10.85	NA	32	32
**Amphotericin B**	0.35	NT	NT	NT
**Fluconazole**	NT	0.25	2	8

^a^ Exhibited with IC_50_ value (μg/mL); ^b^ Exhibited with MIC (μg/mL); NT = Not tested; NA = Not active.

**Table 3:** Cytotoxic and PTP1B inhibitory activitives of woodylides A–C (**1–3**).

Compound	Cytotoxicity (IC_50_, μg/mL)					PTP1B Inhibitory Activity (IC_50_, μg/mL)
	HCT-116	A549	HeLa	QGY-7703	MDA231	
**1**	NT	37.83	11.22	25.80	NA	NT
**2**	NT	NT	NT	NT	NT	NT
**3**	9.4	NA	NA	NA	NT	4.7
**Sodium orthovanadate**	NT	NT	NT	NT	NT	88.46

NT = Not tested; NA = Not active.

## 3. Experimental Section

### 3.1. General Experimental Procedures

Optical rotations were determined with a Perkin-Elmer 341 polarimeter with 1 mm cell. IR spectra were recorded on a Bruker vector 22 spectrometer with KBr pellets. The NMR experiments were conducted on Bruker AVANCE-600 and Bruker AMX-500 instruments. HRESIMS and ESIMS were obtained on a Q-Tof micro YA019 mass spectrometer. In antifungal evaluation, IC_50_ values were calculated on XL*fit* 4.2 software (IDBS: Alameda, CA, USA, 2005). Reversed-phase HPLC was performed on YMC-Pack Pro C_18_ RS (5 μm) columns with a Waters 1525/2998 liquid chromatograph. Column chromatographies were carried out on silica gel 60 (200–300 mesh; Yantai, China), Sephadex LH-20 (Amersham Biosciences). TLC was carried out using HSGF 254 plates and visualized by spraying with anisaldehyde-H_2_SO_4_ reagent. 

### 3.2. Animal Material

The sponge, identified by Jin-He Li (Institute of Oceanology, Chinese Academy of Sciences, China), was collected off Woody (Yongxing) Island and seven connected islets in the South China Sea in June 2007. A voucher sample (No. B-3) was deposited in the Laboratory of Marine Drugs, Department of Pharmacy, Changzheng Hospital, Second Military Medical University, China. 

### 3.3. Extraction and Isolation

The air-dried and powdered sponge (1.0 kg, dry weight) was extracted with 95% aqueous EtOH, and the combined extracts were concentrated under reduced pressure at 45 °C to yield the crude extract (100 g). This extract was suspended in H_2_O and extracted with EtOAc and *n*-BuOH to afford the EtOAc- and *n*-BuOH-soluble extracts. The EtOAc-soluble extract (80 g) was partitioned between 90% aqueous MeOH and *n*-hexane to afford the *n*-hexane-soluble extract (21 g), which was subjected to Vacuum Liquid Chromatography (VLC) on silica gel by gradient elution using *n*-hexane/acetone (100:1, 50:1, 20:1, 15:1, 10:1, 5:1, 1:1, 0:1) as solvents to give seven subfractions (A–G). Subfraction G was subjected to CC on Sephadex LH-20, ODS and further purified by reversed-phase preparative HPLC (YMC-Pack Pro C_18_ *RS*, 5 μm, 10 × 250 mm, 2.0 mL/min), to yield compound **1** (CH_3_OH/H_2_O 80:20, 2.0 mL/min, 208 nm, *t*_R_ = 44.03 min, 10.2 mg), compound **2** (CH_3_OH/H_2_O 80:20, 2.0 mL/min, 201 nm, *t*_R_ = 59.65 min, 2.5 mg), and compound **3** (CH_3_OH:H_2_O 80:20, 2.0 mL/min, 208 nm, *t*_R_ = 33.08 min, 22.3 mg). 

Preparation of MTPA esters **1a** and **1b**: Woodylide A (**1**; 1.2 mg (3.8 μmol) and 1.0 mg (3.2 μmol), respectively) was reacted with *R*-(−)- or *S*-(+)-MTPACl (59.4 μmol) in freshly distilled dry pyridine (500 µL) and stirred under N_2_ at room temperature for 18 h, respectively, and then the solvent was removed. The products were purified by mini-CC on silica gel (200 mesh, *n*-hexane:EtOAc, 3:1) to afford *S*-(−)- and *R*-(+)-MTPA esters **1a** and **1b**, respectively.

Preparation of MTPA esters **3a** and **3b**: Woodylide C (**3**; 1.2 mg (4.0 μmol) and 1.1 mg (3.7 μmol), respectively) was similarly processed to give *S*-(−)- and *R*-(+)- MTPA esters **3a** and **3b**, respectively.

Woodylide A (**1**): Colorless oil; [α]^22^_D_ −15.0 (*c* 0.06, MeOH); IR (KBr) *ν*_max_ 3275, 2961, 2928, 2874, 2858, 1742, 1650, 1460, 1438, 1412, 1376, 1356, 1286, 1252, 1213, 1169, 1108, 1066, 1036, 1016, 992, 966, 933, 870, 852, 806, 781, 706 cm^−1^; ^1^H NMR (CDCl_3_, 500 MHz) and ^13^C NMR (CDCl_3_, 125 MHz) data, see Table 1; HRESIMS *m/z* 337.2354 [M + Na]^+^ (calcd for C_18_H_34_O_4_Na, 337.2355). CD spectrum (*c* 1.91 × 10^−3^ M, CH_3_CN), 197 nm (Δ*ε* 3.27), 200 nm (Δ*ε* 3.54).

Woodylide B (**2**): Colorless oil; [α]^22^_D_ +5.5 (*c* 0.06, MeOH); IR (KBr) *ν*_max_ 3301, 2961, 2928, 2874, 2857, 1742, 1667, 1462, 1438, 1410, 1378, 1358, 1286, 1169, 1108, 1067, 1035, 1018, 994, 872, 852, 806, 781, 706 cm^−1^; ^1^H NMR (CDCl_3_, 600 MHz) and ^13^C NMR (CDCl_3_,150 MHz) data, see Table 1; HRESIMS *m/z* 351.2513 [M + Na]^+^ (calcd for C_19_H_36_O_4_Na, 351.2511). CD spectrum (*c* 2.13 × 10^−3^ M, CH_3_CN), 196 nm (Δ*ε* 3.02), 203 nm (Δ*ε* 2.36). 

Woodylide C (**3**): Light yellow oil; [α]^22^_D_ −11.4 (*c* 0.14, MeOH); IR (KBr) *ν*_max_ 3422, 2961, 2928, 2874, 2858, 1757, 1655, 1462, 1401, 1379, 1342, 1285, 1252, 1209, 1169, 1109, 1063, 1021, 954, 915, 879, 801, 729, 666 cm^−1^; ^1^H NMR (CDCl_3_, 600 MHz) and ^13^C NMR (CDCl_3_, 150 MHz) data, see Table 1; HRESIMS *m/z* 323.2200 [M + Na]^+^ (calcd for C_17_H_32_O_4_Na, 323.2198). CD spectrum (*c* 2.32 × 10^−3^ M, CH_3_CN), 191 nm (Δ*ε* 3.23), 196 nm (Δ*ε* 4.72), 202 nm (Δ*ε* 3.61).

^1^H NMR data of **1a** (CDCl_3_, 600 MHz): δ 2.59 (1H, dd, H-2a), 2.91 (1H, dd, H-2b), 5.27 (1H, s, H-5), 1.47 (1H, dd, H-7a), 1.53 (1H, dd, H-7b), 1.60 (1H, m, H-8), 1.14 (2H, m, H-9), 1.26 (2H, m, H-10), 1.26 (2H, m, H-11), 0.88 (3H, t, H-12), 2.05 (2H, m, H-13), 1.01 (3H, t, H-14), 1.56 (2H, m, H-15), 0.86 (3H, t, H-16), 0.94 (3H, d, H-17), 3.60 (3H, s, H-18). 

^1^H NMR data of **1b** (CDCl_3_, 600 MHz): δ 2.59 (1H, dd, H-2a), 2.93 (1H, dd, H-2b), 5.22 (1H, s, H-5), 1.49 (1H, dd, H-7a), 1.53 (1H, dd, H-7b), 1.61 (1H, m, H-8), 1.19 (2H, m, H-9), 1.26 (2H, m, H-10), 1.26 (2H, m, H-11), 0.88 (3H, t, H-12), 1.84 (2H, m, H-13), 0.90 (3H, t, H-14), 1.56 (2H, m, H-15), 0.85 (3H, t, H-16), 0.99 (3H, d, H-17), 3.67 (3H, s, H-18). 

^1^H NMR data of **3a** (CDCl_3_, 600 MHz): δ 2.41 (1H, dd, H-2a), 2.95 (1H, dd, H-2b), 5.04 (1H, s, H-5), 1.29 (1H, dd, H-7a), 1.73 (1H, dd, H-7b), 1.80 (1H, m, H-8), 1.11 (2H, m, H-9), 1.73 (2H, m, H-10), 1.27 (2H, m, H-11), 0.81 (3H, t, H-12), 2.04 (2H, m, H-13), 0.98 (3H, t, H-14), 1.27 (2H, m, H-15), 0.79 (3H, t, H-16), 0.90 (3H, d, H-17). 

^1^H NMR data of **3b** (CDCl_3_, 600 MHz): δ 2.50 (1H, dd, H-2a), 3.00 (1H, dd, H-2b), 5.03 (1H, s, H-5), 1.28 (1H, dd, H-7a), 1.76 (1H, dd, H-7b), 1.76 (1H, m, H-8), 1.11 (2H, m, H-9), 1.71 (2H, m, H-10), 1.26 (2H, m, H-11), 0.81 (3H, t, H-12), 1.95 (2H, m, H-13), 0.97 (3H, t, H-14), 1.26 (2H, m, H-15), 0.78 (3H, t, H-16), 0.89 (3H, d, H-17).

### 3.4. Antifungal Evaluation

Antifungal IC_50_ values of woodylides A–C against *C. neoformans* were calculated as described by Ikhlas A. Khan *et al.* [20]. Amphotericin B was used as the positive control. Minimal Inhibition Concentration (MIC) values of woodylides A–C were determined against three indicators (*C. albicans*, *T. rubrum*, and *M. gypseum*), following the National Center for Clinical Laboratory Standards (NCCLS) methods [21,22]. Fluconazole was used as the positive control. Briefly, samples (dissolved in DMSO) were serially diluted in 20% DMSO/saline and transferred (10 µL) in duplicate to 96 well flat bottom microplates. Bacterial strains were grown aerobically at 30 °C in SDA for 16–20 h. A set of different concentrations of compounds **1–3** prepared in RPMI 1640 were next inoculated with the microorganisms and incubated 70–74 h for *C. neoformans* at 35 °C, 46 h for *C. albicans* at 35 °C, and 4–7 days for *T. rubrum* and *M. gypseum* at 30 °C. The IC_50_ values were calculated by using the fit model 201 of XL*fit* 4.2 software. The MIC values were evaluated in triplicate for each compound (within the range 1.25–640 μg/mL). 

### 3.5. Cytotoxicity Assay

The cytotoxicity of compounds **1–3** against HCT-116, A549, HeLa, QGY-7703, and MDA231 cell lines was evaluated by the MTT assay as described in a previous report [23]. Briefly, compounds were solubilized in DMSO with the working concentration of test substances ranging from 1 to 100 μg/mL. Cells at the exponential growth phase were harvested and seeded into 96-well plates. After incubation for 24 h, the cells were treated with various concentrations of test substances for 48 h and then incubated with 1 mg/mL MTT at 37 °C for 4 h, followed by dissolving in DMSO. The produced formazan was measured by the absorbance at 570 nm on a microplate reader. The IC_50_ values were calculated on the basis of percentage inhibition using the linear regression method.

### 3.6. PTP1B Inhibitory Assay

PTP1B inhibitory activity was determined using a PTP1B inhibitory assay as described previously [24]. The enzymatic activities of the PTP1B catalytic domain were determined at 30 °C by monitoring the hydrolysis of *p*NPP. Dephosphorylation of *p*NPP generated the product *p*NP, which was monitored at an absorbance of 405 nm. In a typical 100 μL assay mixture containing 50 mmol/L 3-[*N*-morpholino]propanesulfonic acid (MOPs), pH 6.5, 2 mmol/L *p*NPP, and 30 nmol/L recombinant PTP1B, activities were continuously monitored and the initial rate of the hydrolysis was determined using the early linear region of the enzymatic reaction kinetic curve. 

## 4. Conclusions

In this paper we report the isolation and the structural determination of three new linear polyketides, woodylides A–C, endowed with antifungal, antineoplastic, and PTP1B inhibitory activities, from the South China Sea marine sponge *P. simplex.* Unfortunately, due to the lack of compound **2**, the absolute configuration at C-3 as well as the bioactivity of woodylide B could not be determined. Woodylide C exhibited a good PTP1B inhibitory activity, and deserves further study for its therapeutic potential against type II diabetes and obesity diseases. 

## Supplementary Files

Supplementary File 1: PDF-Document (PDF, 3766 KB)
