# Supplementary material for: Woodylides A–C, New Cytotoxic Linear Polyketides from the South China Sea Sponge Plakortis simplex
Source: Mar Drugs. 2012 May 7;10(5):1027–36. doi: 10.3390/md10051027 (PMC3397464; doi:10.3390/md10051027)

Supporting Information

S1. ^{1}H NMR spectrum of woodylide A (**1**) in $CDCl_3$.
S2. ^{13}C NMR spectrum of woodylide A (**1**) in $CDCl_3$.
S3. DEPT spectrum of woodylide A (**1**) in $CDCl_3$.
S4. HSQC spectrum of woodylide A (**1**) in $CDCl_3$
S5. HMBC spectrum of woodylide A (**1**) in $CDCl_3$.
S6. ^{1}H–^{1}H COSY spectrum of woodylide A (**1**) in $CDCl_3$.
S7. NOESY spectrum of woodylide A (**1**) in $CDCl_3$.
S8. IR spectrum of woodylide A (**1**).
S9. HRESIMS of woodylide A (**1**).
S10. ^{1}H NMR spectrum of woodylide B (**2**) in $CDCl_3$.
S11. ^{13}C NMR spectrum of woodylide B (**2**) in $CDCl_3$.
S12. DEPT spectrum of woodylide B (**2**) in $CDCl_3$.
S13. HSQC spectrum of woodylide B (**2**) in $CDCl_3$
S14. HMBC spectrum of woodylide B (**2**) in $CDCl_3$.
S15. ^{1}H-^{1}H COSY spectrum of woodylide B (**2**) in $CDCl_3$.
S16. NOESY spectrum of woodylide B (**2**) in $CDCl_3$.
S17. IR spectrum of woodylide B (**2**).
S18. HRESIMS of woodylide B (**2**).
S19. ^{1}H NMR spectrum of woodylide C (**3**) in $CDCl_3$.
S20. ^{13}C NMR spectrum of woodylide C (**3**) in $CDCl_3$.
S21. DEPT spectrum of woodylide C (**3**) in $CDCl_3$.
S22. HSQC spectrum of woodylide C (**3**) in $CDCl_3$
S23. HMBC spectrum of woodylide C (**3**) in $CDCl_3$.
S24. ^{1}H–^{1}H COSY spectrum of woodylide C (**3**) in $CDCl_3$.
S25. NOESY spectrum of woodylide C (**3**) in $CDCl_3$.
S26. IR spectrum of woodylide C (**3**).
S27. HRESIMS of woodylide C (**3**).
S28. CD spectrum of woodylides A–C (**1**–**3**).

S1. ^{1}H NMR spectrum of woodylide A (**1**) in $CDCl_3$.

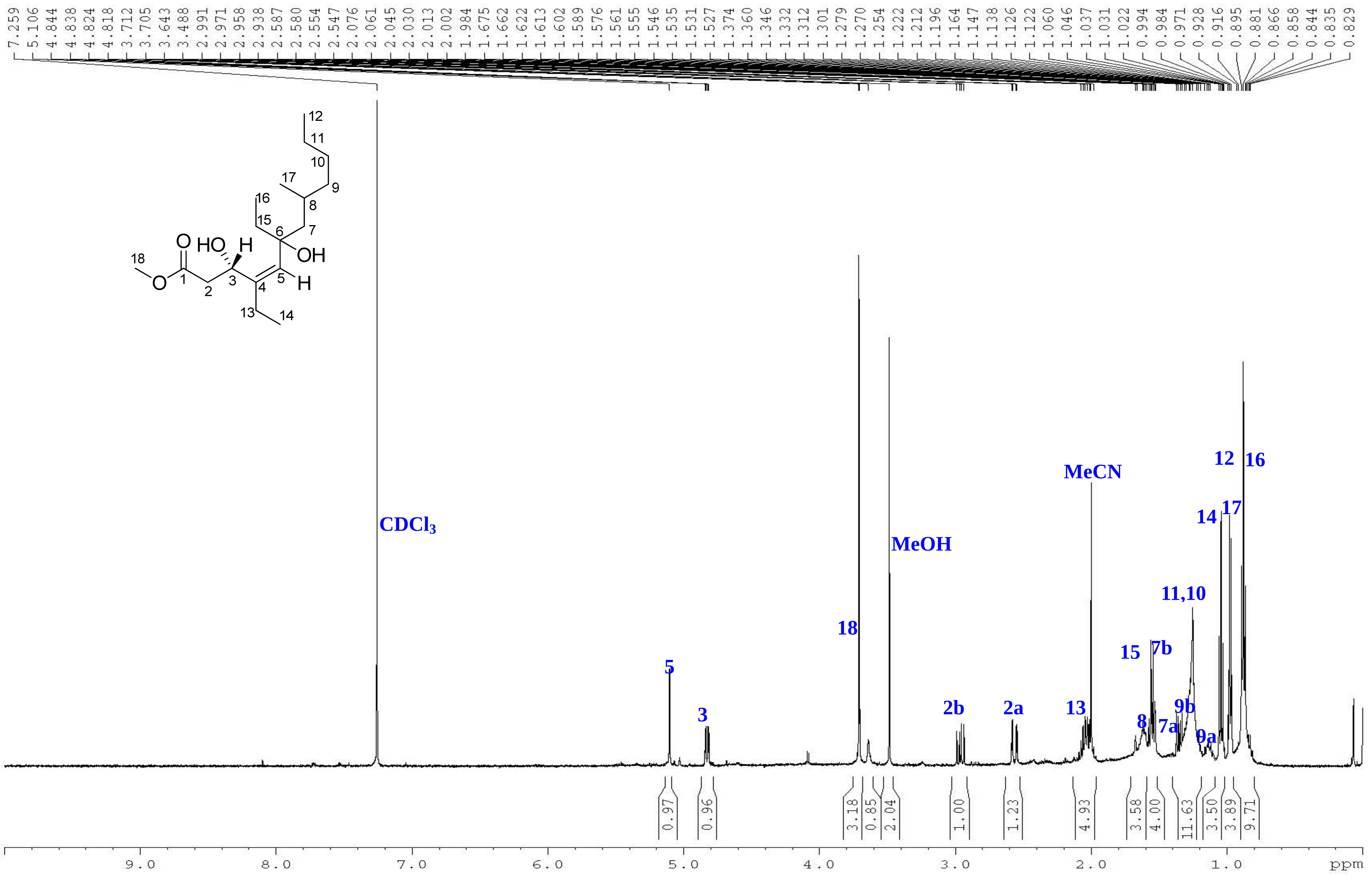

S2. ^{13}C NMR spectrum of woodylide A (**1**) in $CDCl_3$.

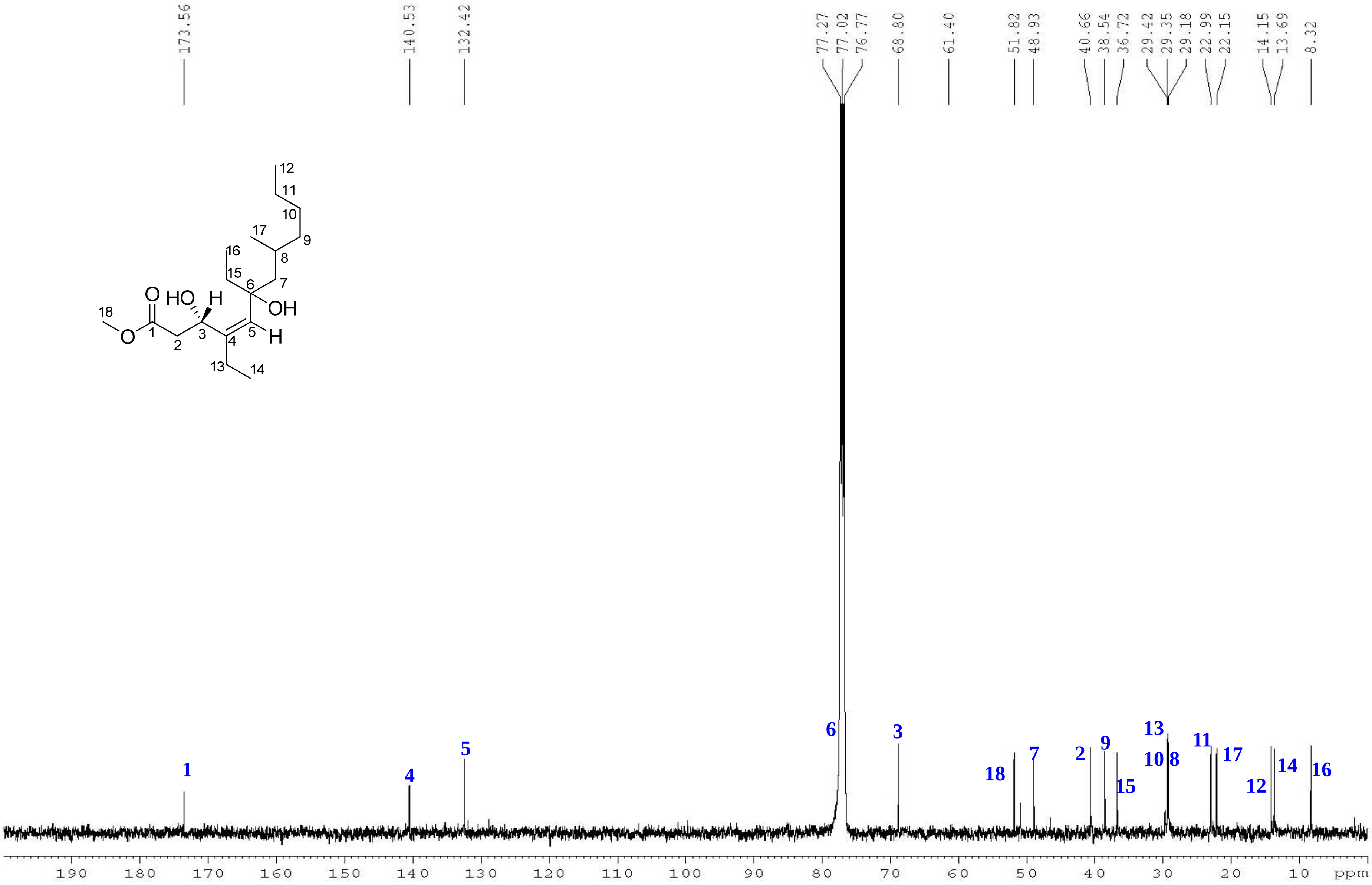

S3. DEPT spectrum of woodylide A (**1**) in $CDCl_3$.

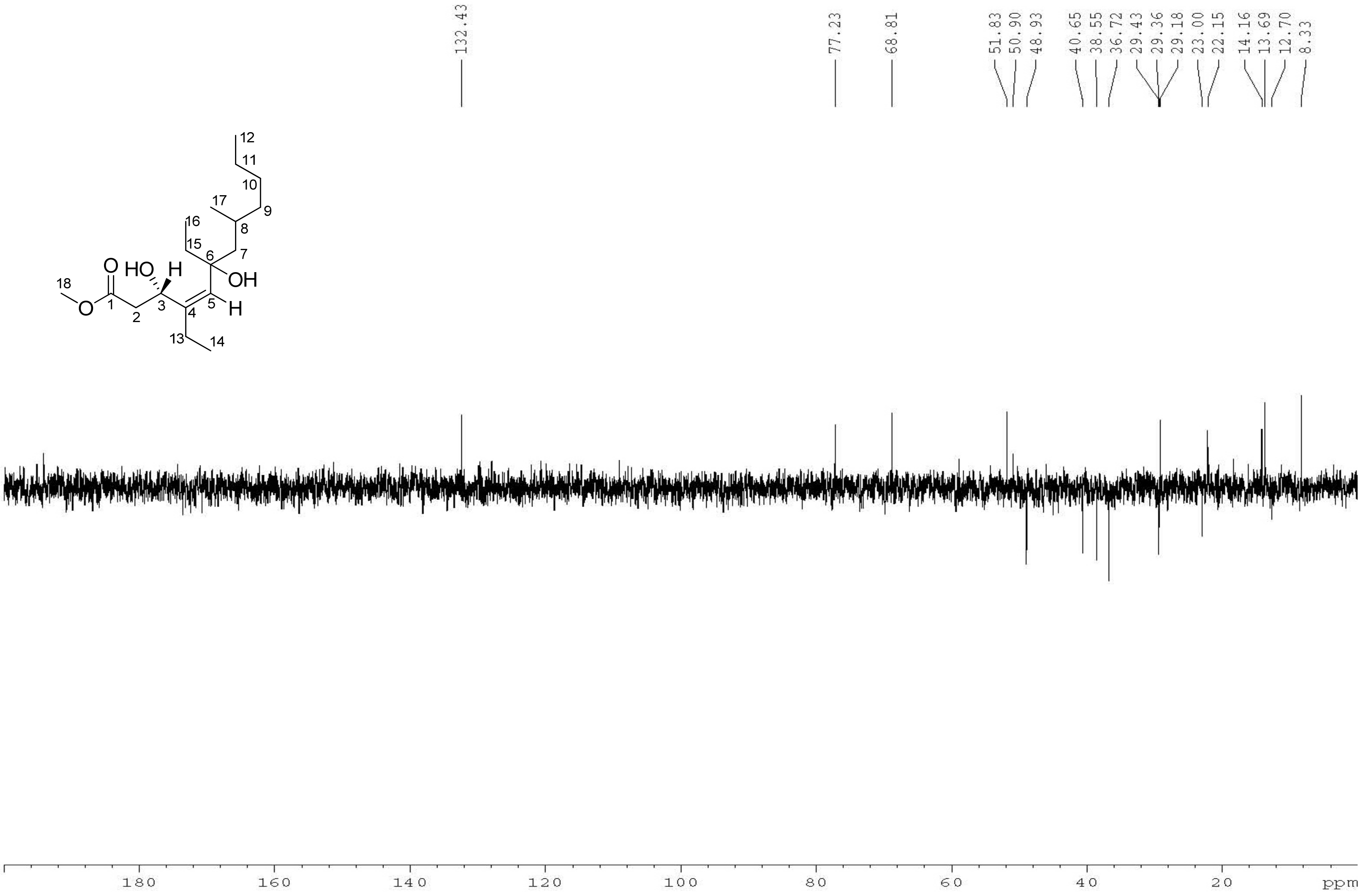

S4. HSQC spectrum of woodylide A (**1**) in $CDCl_3$.

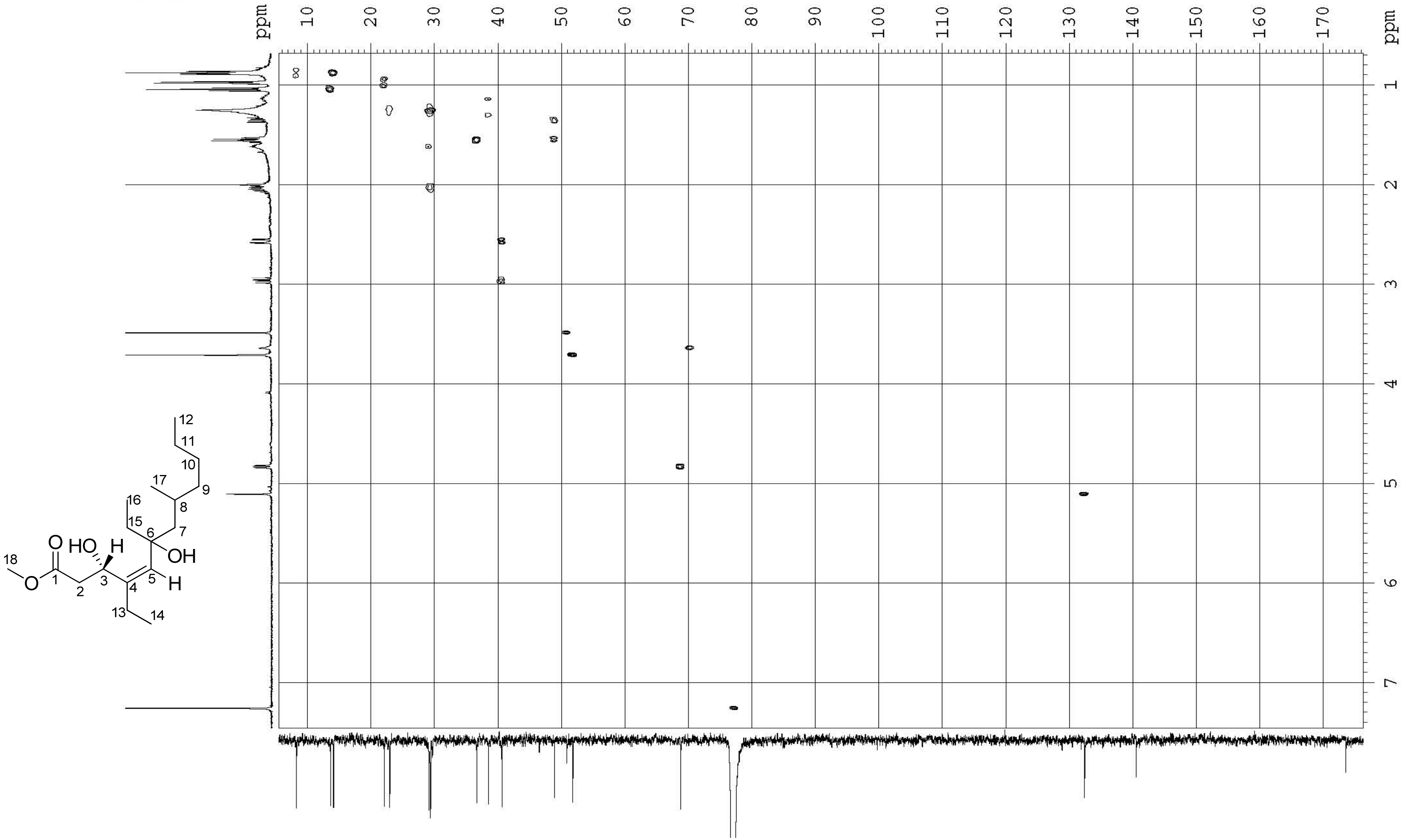

S5. HMBC spectrum of woodylide A (**1**) in $CDCl_3$.

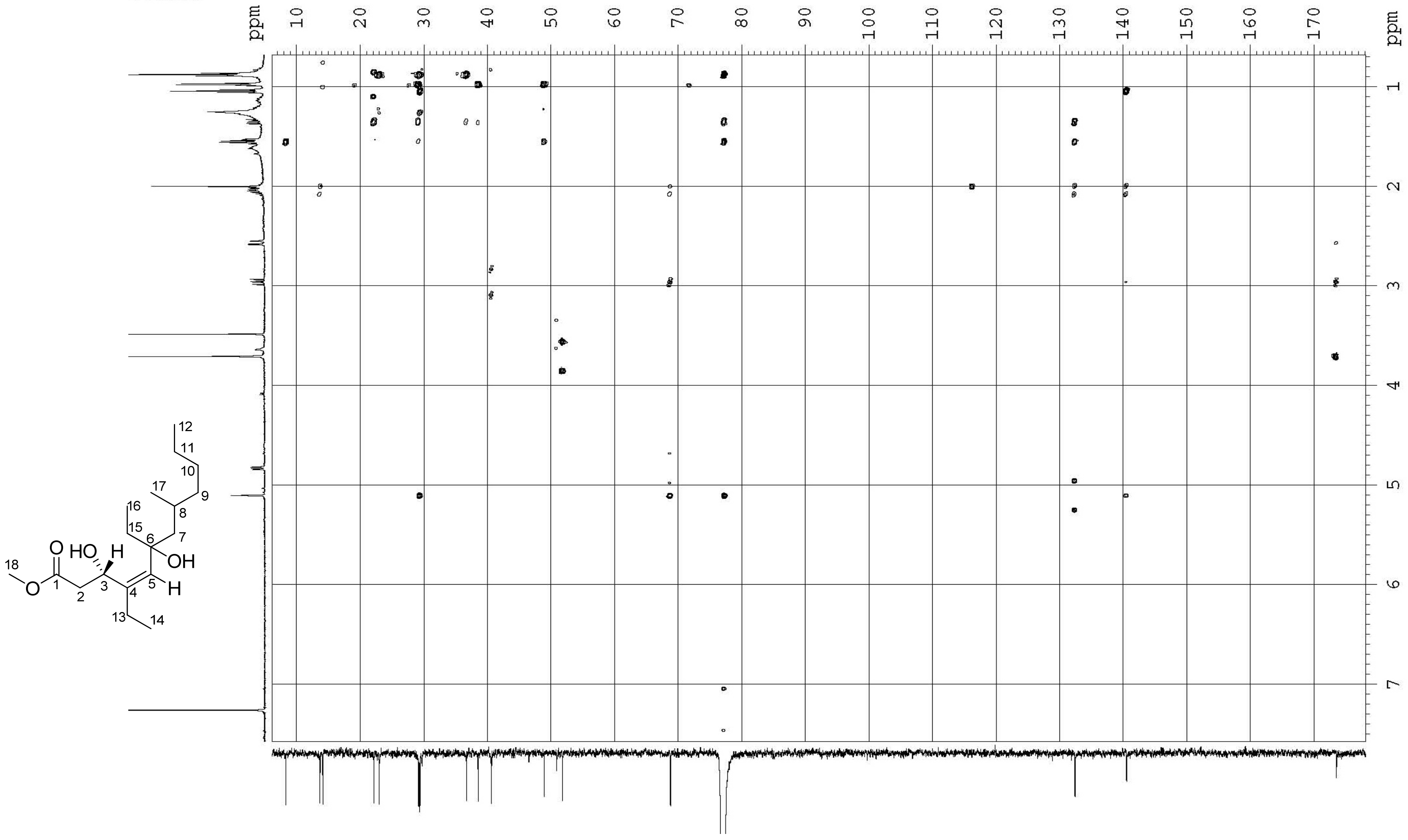

S6. ^{1}H–^{1}H COSY spectrum of woodylide A (**1**) in $CDCl_3$.

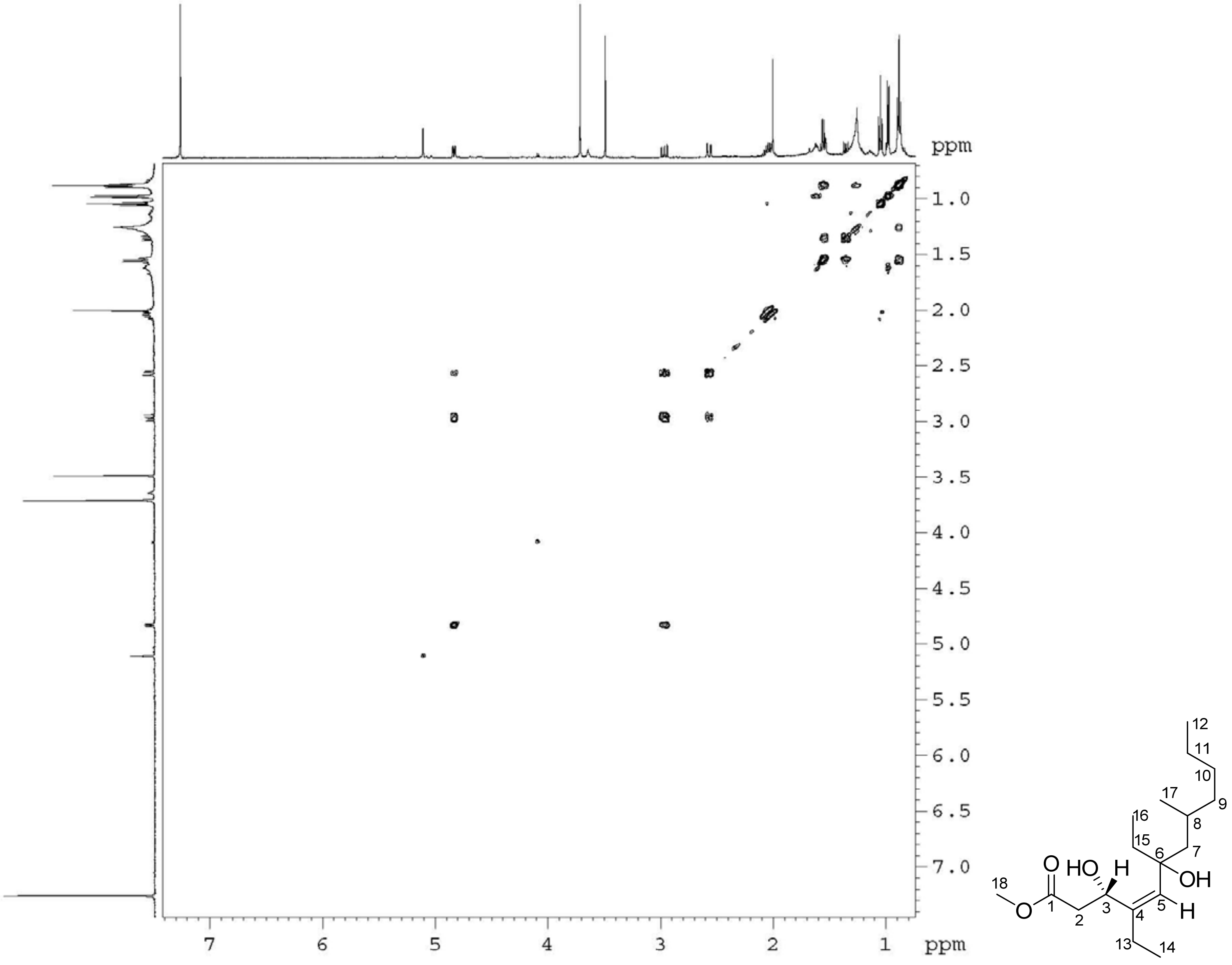

S7. NOESY spectrum of woodylide A (**1**) in $CDCl_3$.

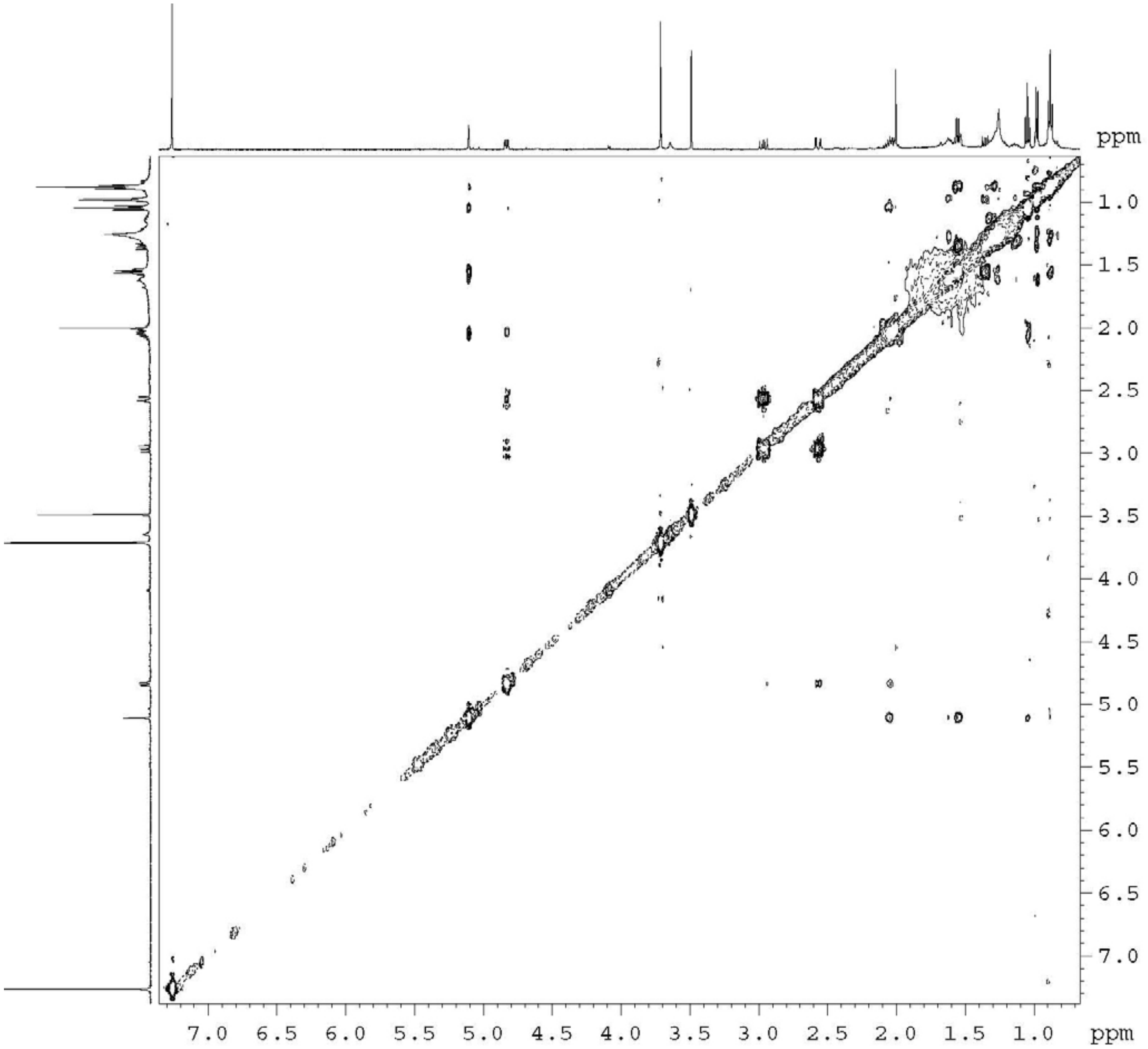

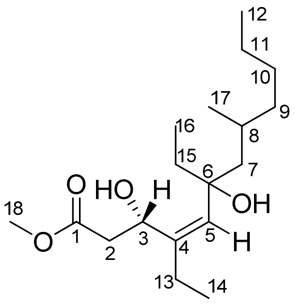

S8. IR spectrum of woodylide A (**1**).

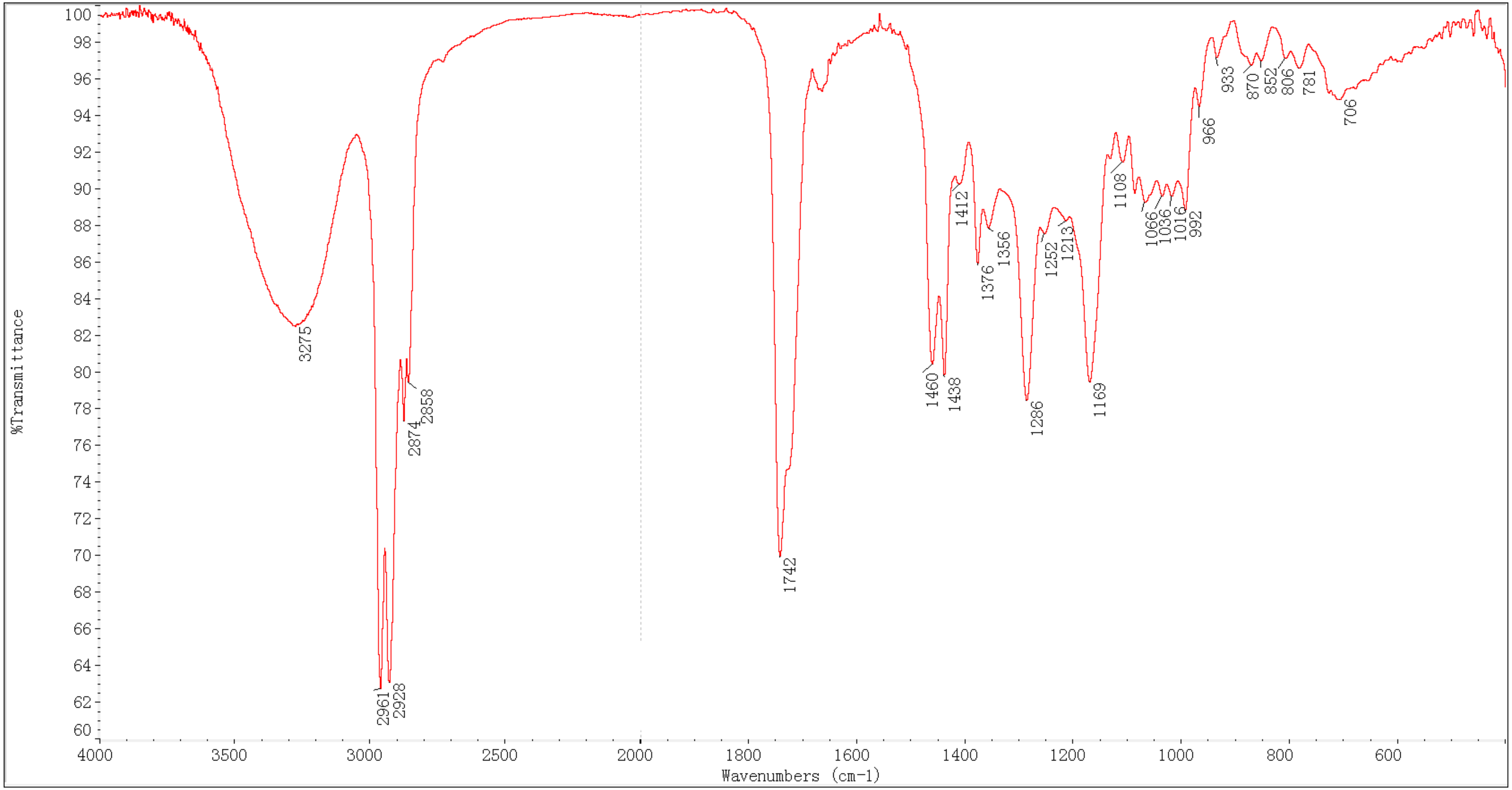

S9. HRESIMS of woodylide A (**1**).

Tolerance = 50.0 PPM / DBE: min = -1.5, max = 50.0
Selected filters: None

Monoisotopic Mass, Even Electron Ions
12 formula(e) evaluated with 1 results within limits (up to 50 closest results for each mass)
Elements Used:
C: 5-20 H: 5-40 O: 1-6 Na: 1-1

SIPI Q-Tof micro 13-Oct-2011,13:33:04
YA019 0.00000000
WQ11-513H1 6 (0.207) AM (Cen,4, 80.00, Ar,5000.0,325.16,0.70); Sm (SG, 2x1.00); Cm (3:11) TOF MS ES+
1.56e5

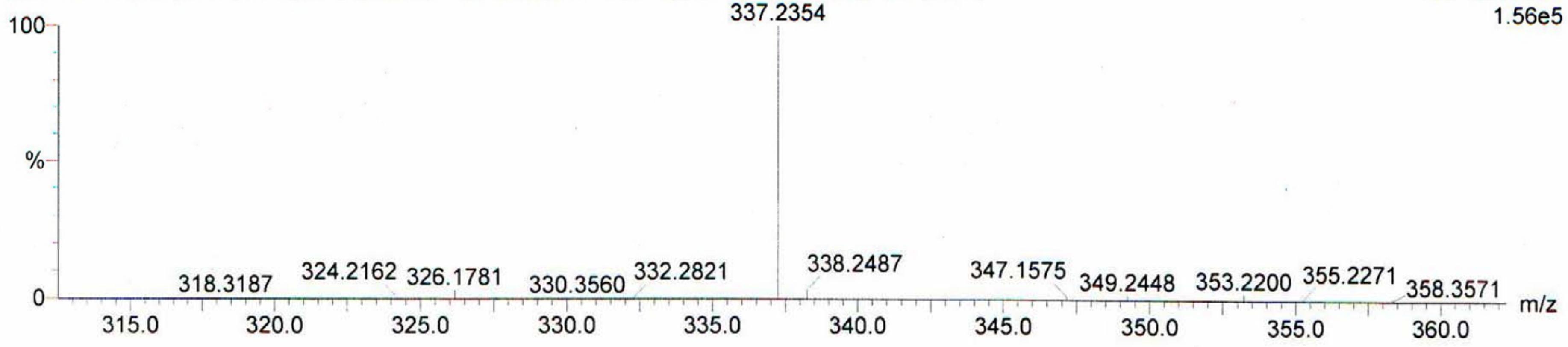

Minimum:	65.00				-1.5		
Maximum:	100.00		5.0	50.0	50.0		
Mass	RA	Calc. Mass	mDa	PPM	DBE	i-FIT	Formula
337.2354	100.00	337.2355	-0.1	-0.3	1.5	45562.2	C18 H34 O4 Na

S10. ^{1}H NMR spectrum of woodylide B (**2**) in $CDCl_3$.

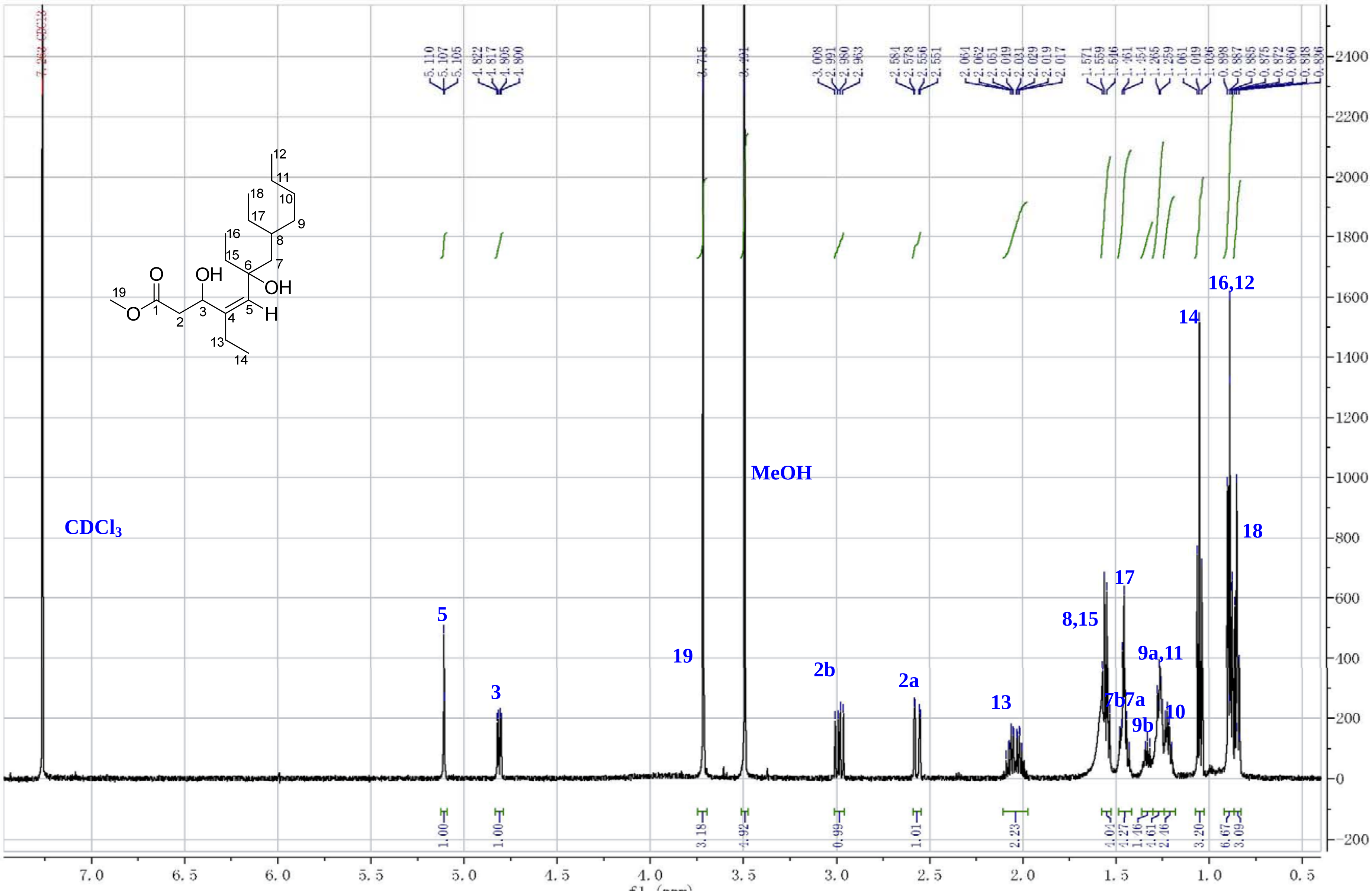

S11. ^{13}C NMR spectrum of woodylide B (**2**) in $CDCl_3$.

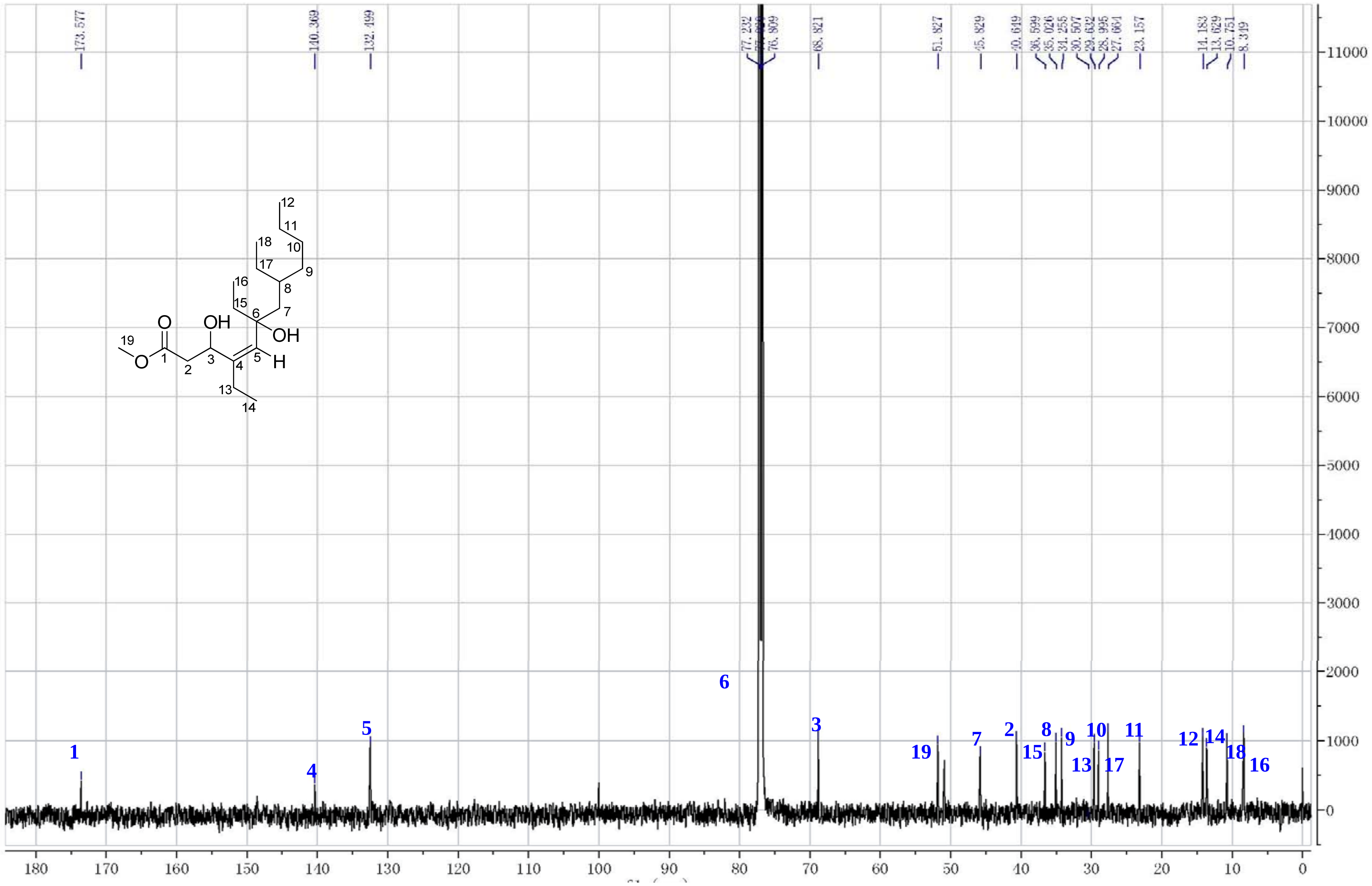

S12. DEPT spectrum of woodylide B (**2**) in $CDCl_3$.

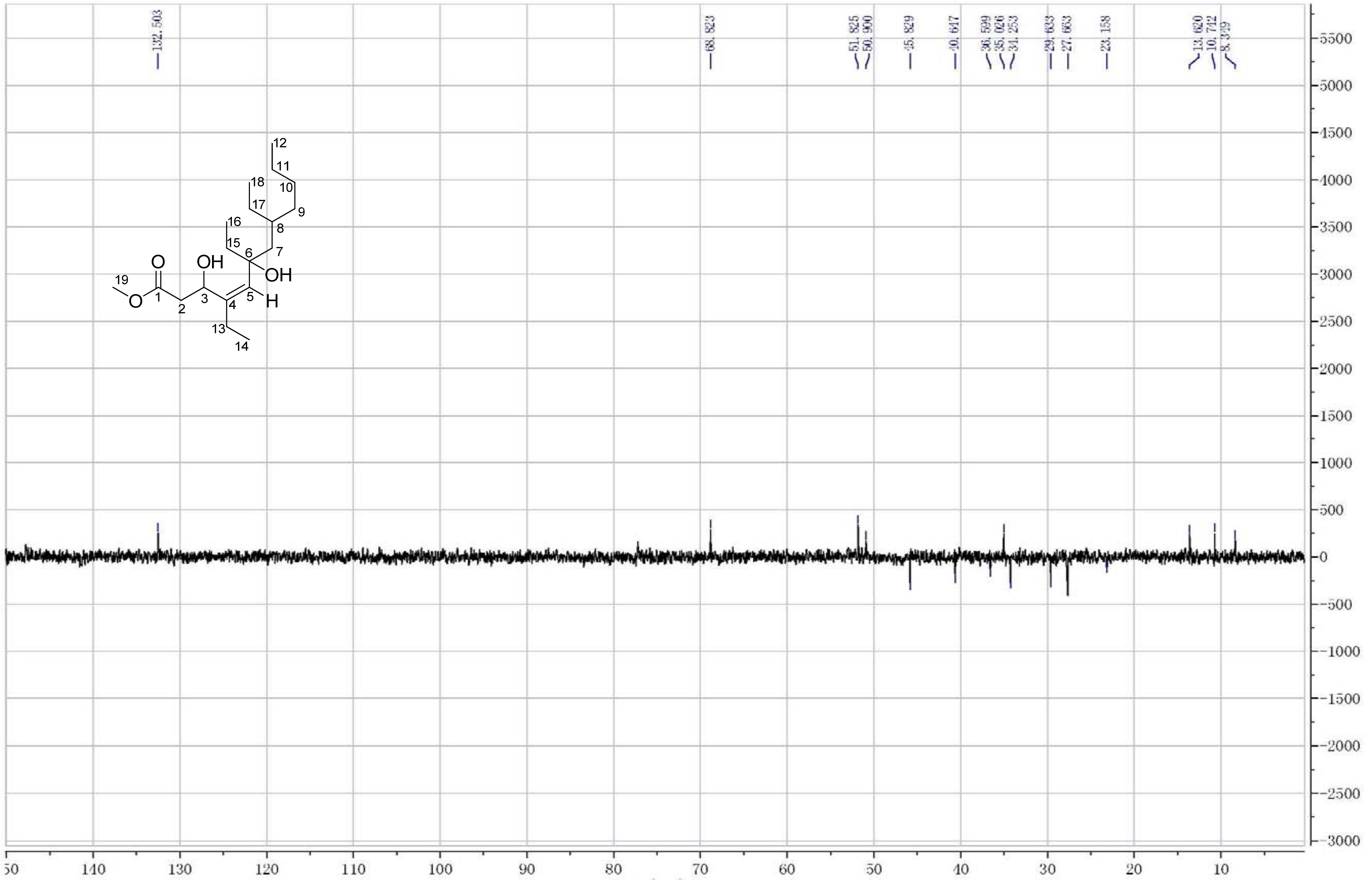

S13. HSQC spectrum of woodylide B (**2**) in $CDCl_3$

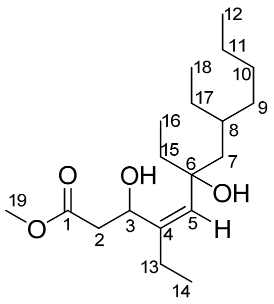

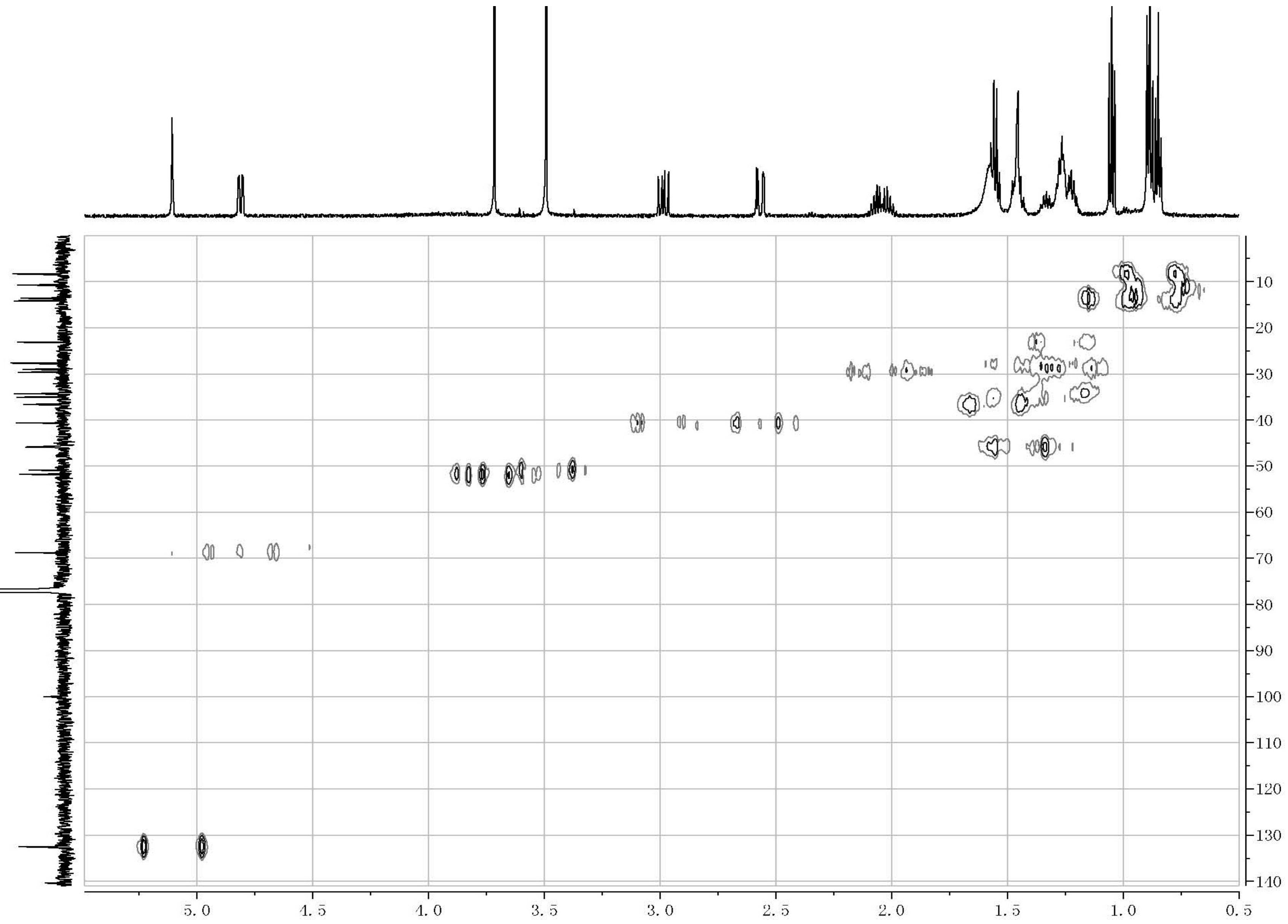

S14. HMBC spectrum of woodylide B (**2**) in $CDCl_3$.

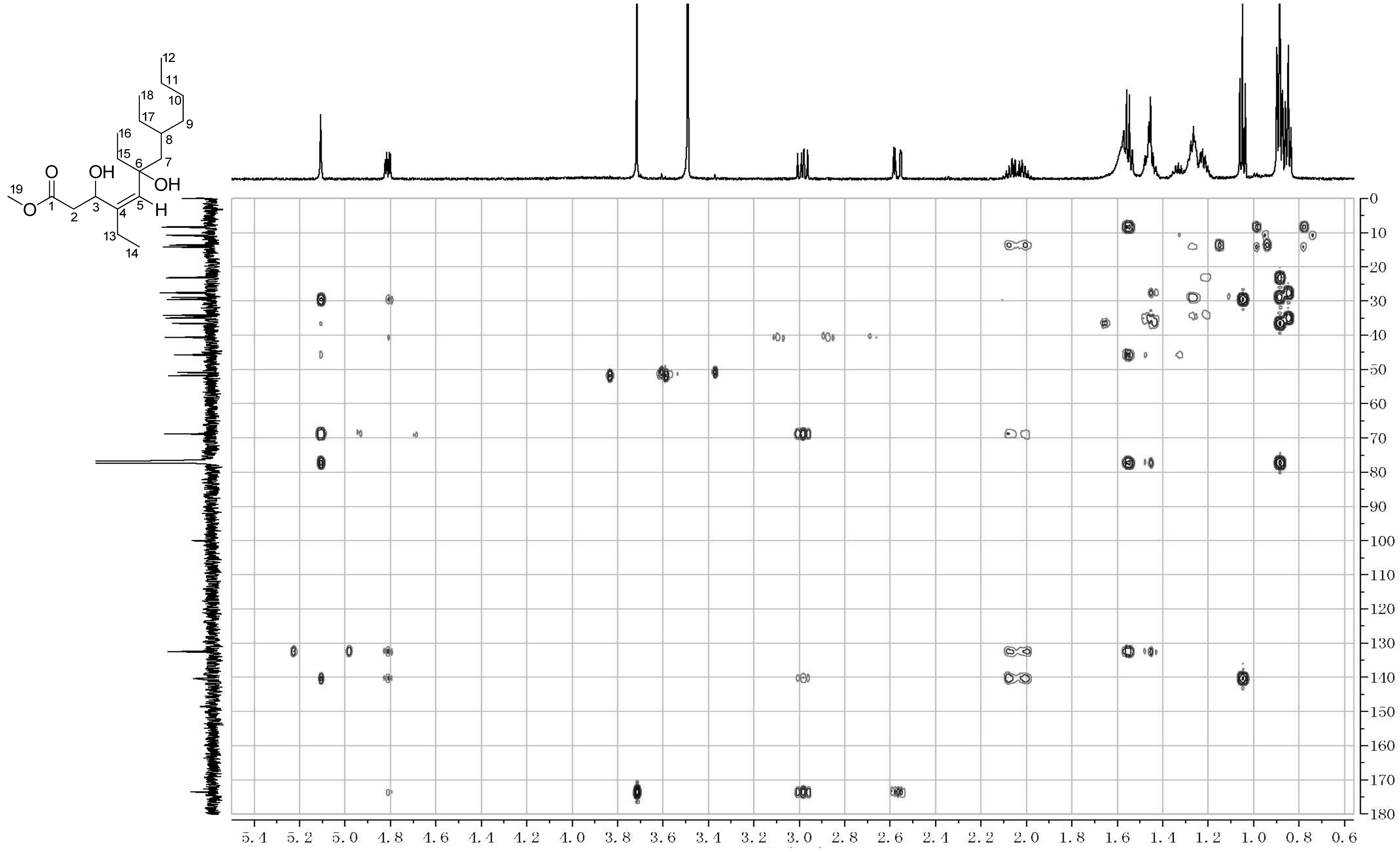

S15. ^{1}H-^{1}H COSY spectrum of woodylide B (**2**) in $CDCl_3$.

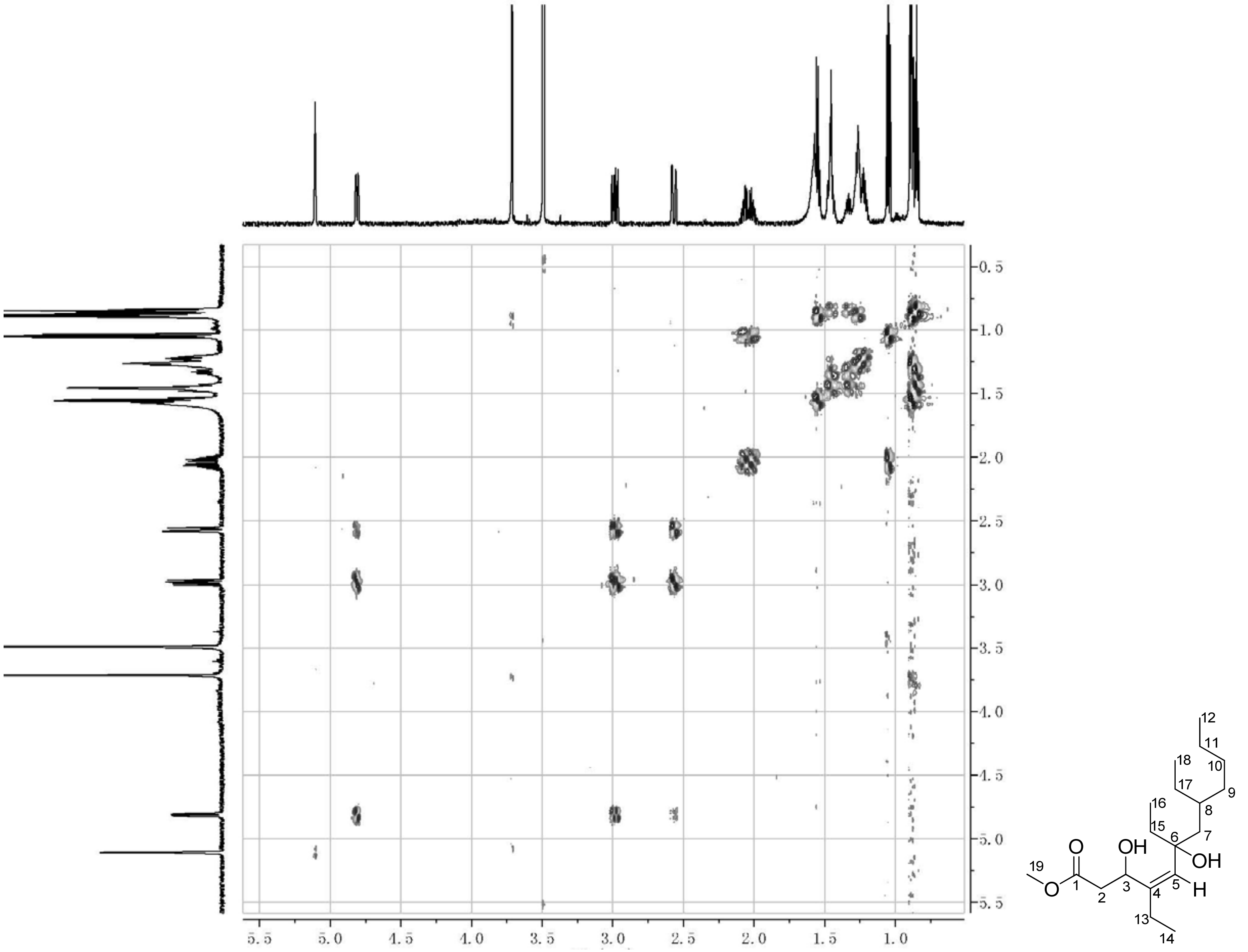

S16. NOESY spectrum of woodylide B (**2**) in $CDCl_3$.

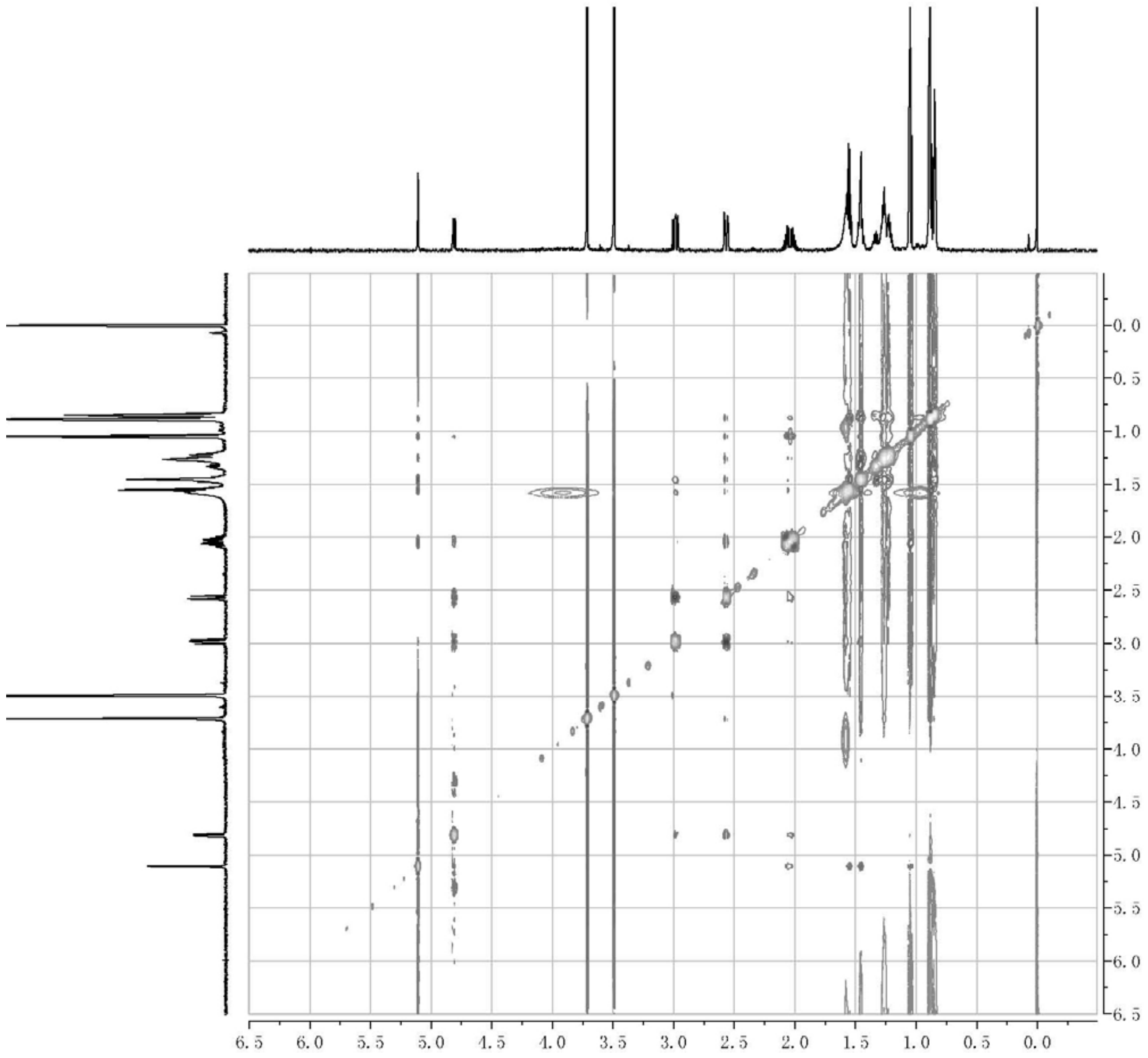

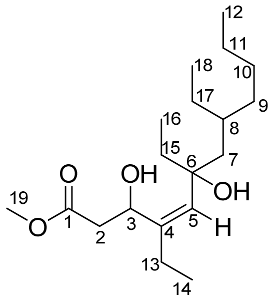

S17. IR spectrum of woodylide B (**2**).

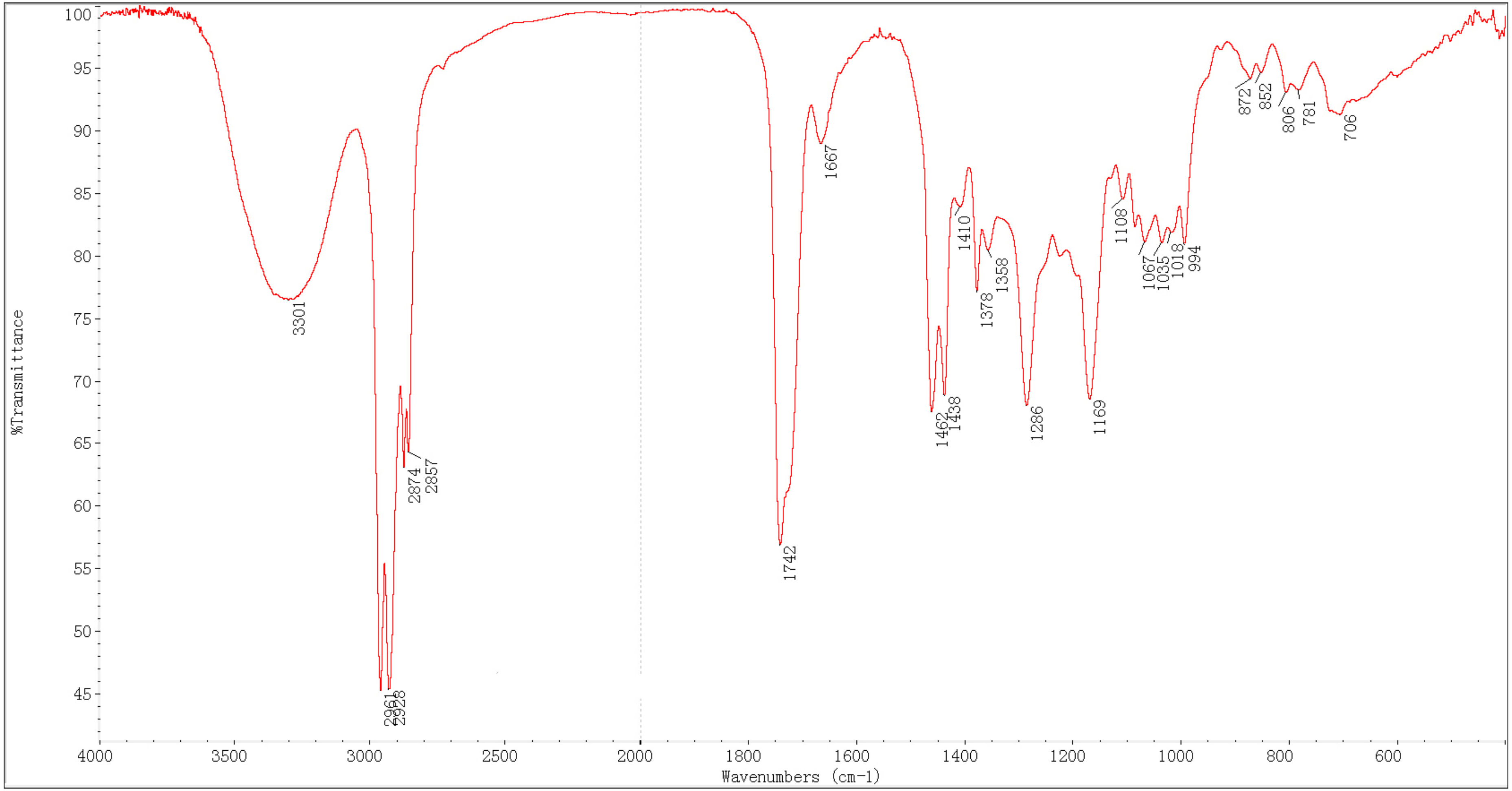

S18. HRESIMS of woodylide B (**2**).

Elemental Composition Report

Tolerance = 50.0 PPM / DBE: min = -1.5, max = 50.0
Selected filters: None

Monoisotopic Mass, Even Electron Ions
11 formula(e) evaluated with 1 results within limits (up to 50 closest results for each mass)
Elements Used:
C: 5-20 H: 5-40 O: 1-6 Na: 1-1

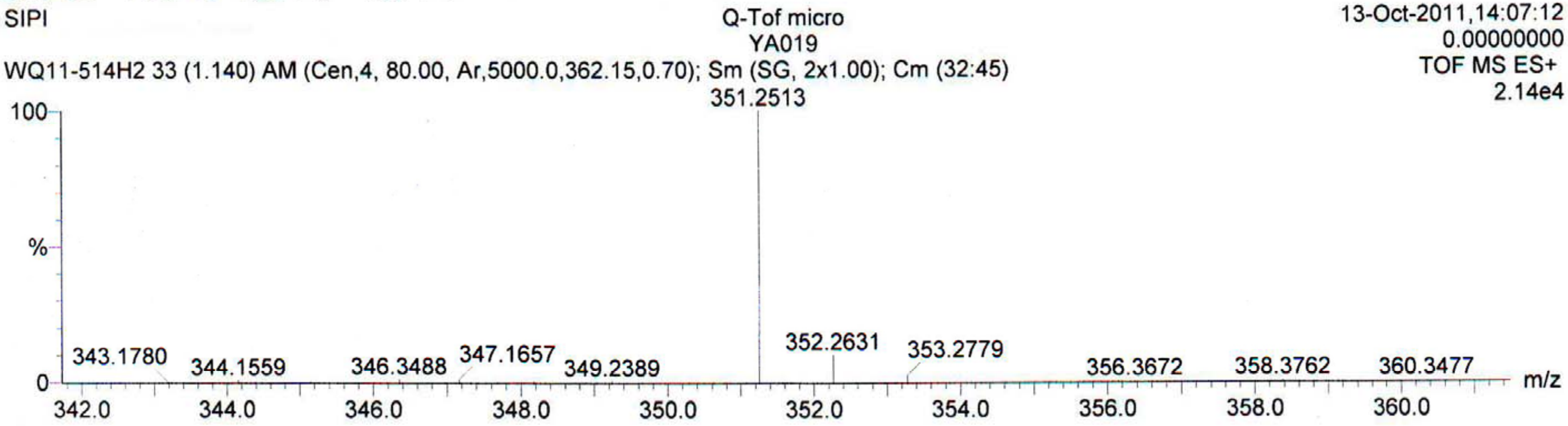

Minimum:	65.00				-1.5		
Maximum:	100.00		5.0	50.0	50.0		

Mass	RA	Calc. Mass	mDa	PPM	DBE	i-FIT	Formula
351.2513	100.00	351.2511	0.2	0.6	1.5	983.6	C19 H36 O4 Na

S19. ^{1}H NMR spectrum of woodylide C (**3**) in $CDCl_3$.

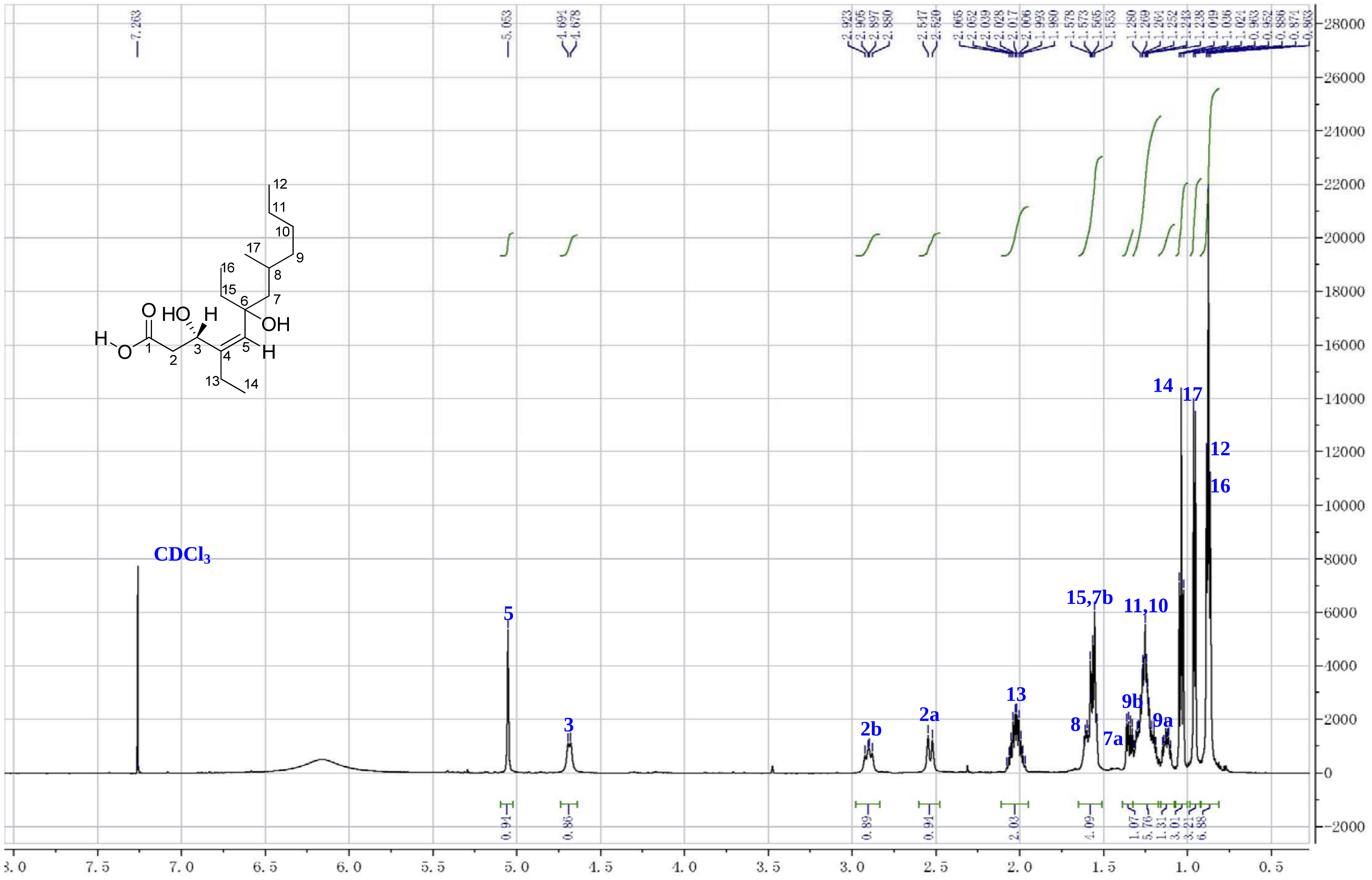

S20. ^{13}C NMR spectrum of woodylide C (**3**) in $CDCl_3$.

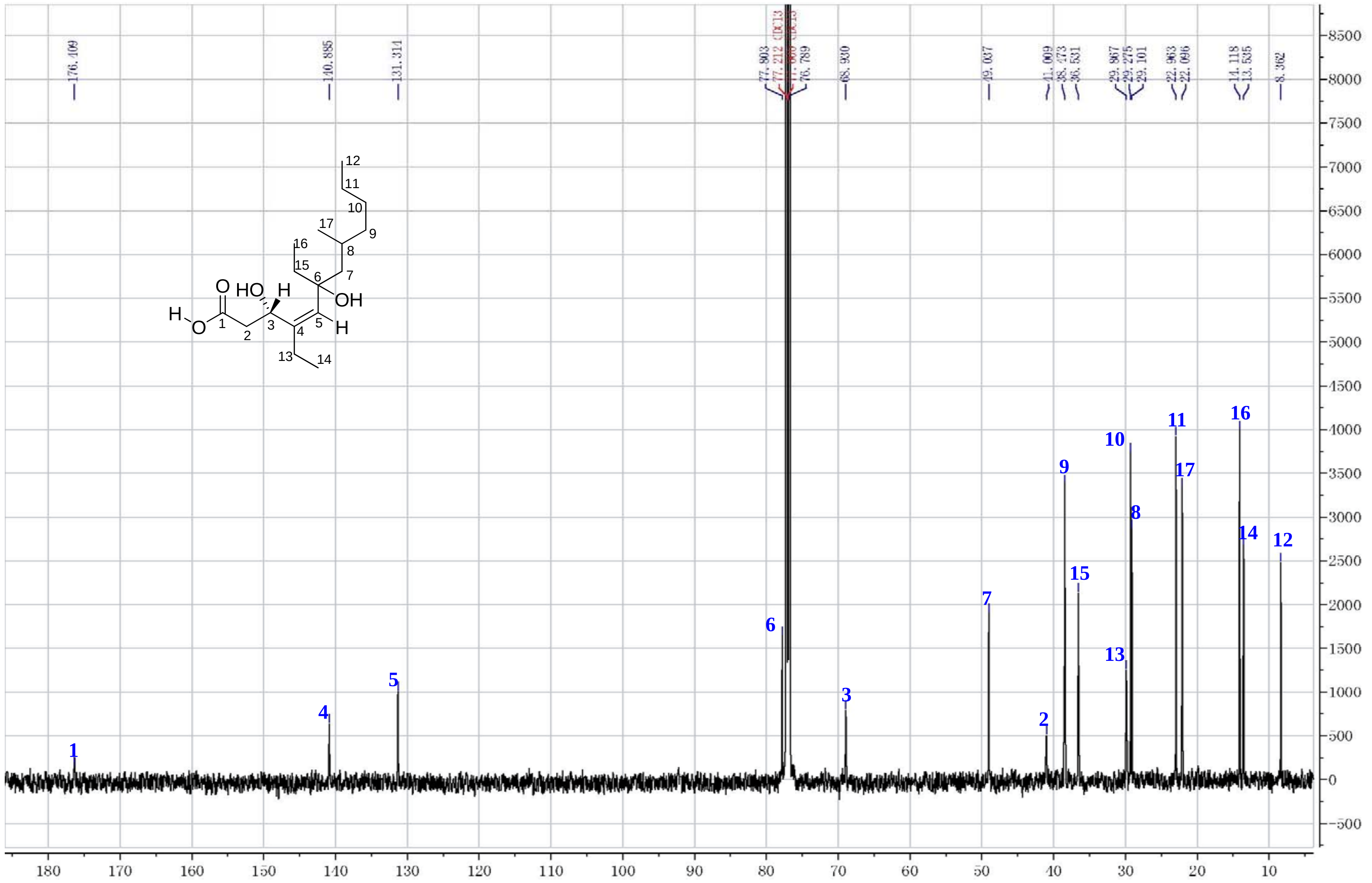

S21. DEPT spectrum of woodylide C (**3**) in $CDCl_3$.

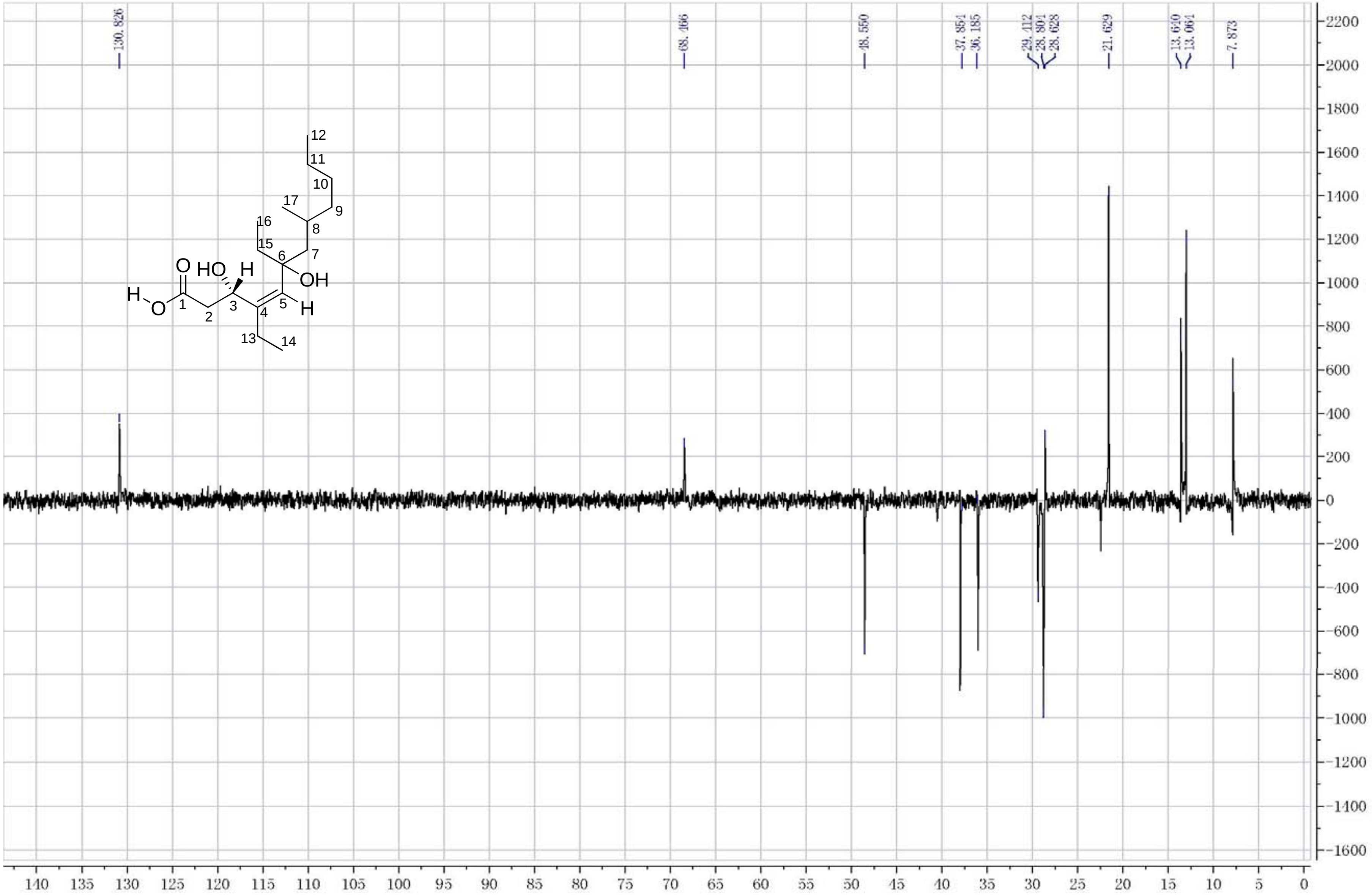

S22. HSQC spectrum of woodylide C (**3**) in $CDCl_3$.

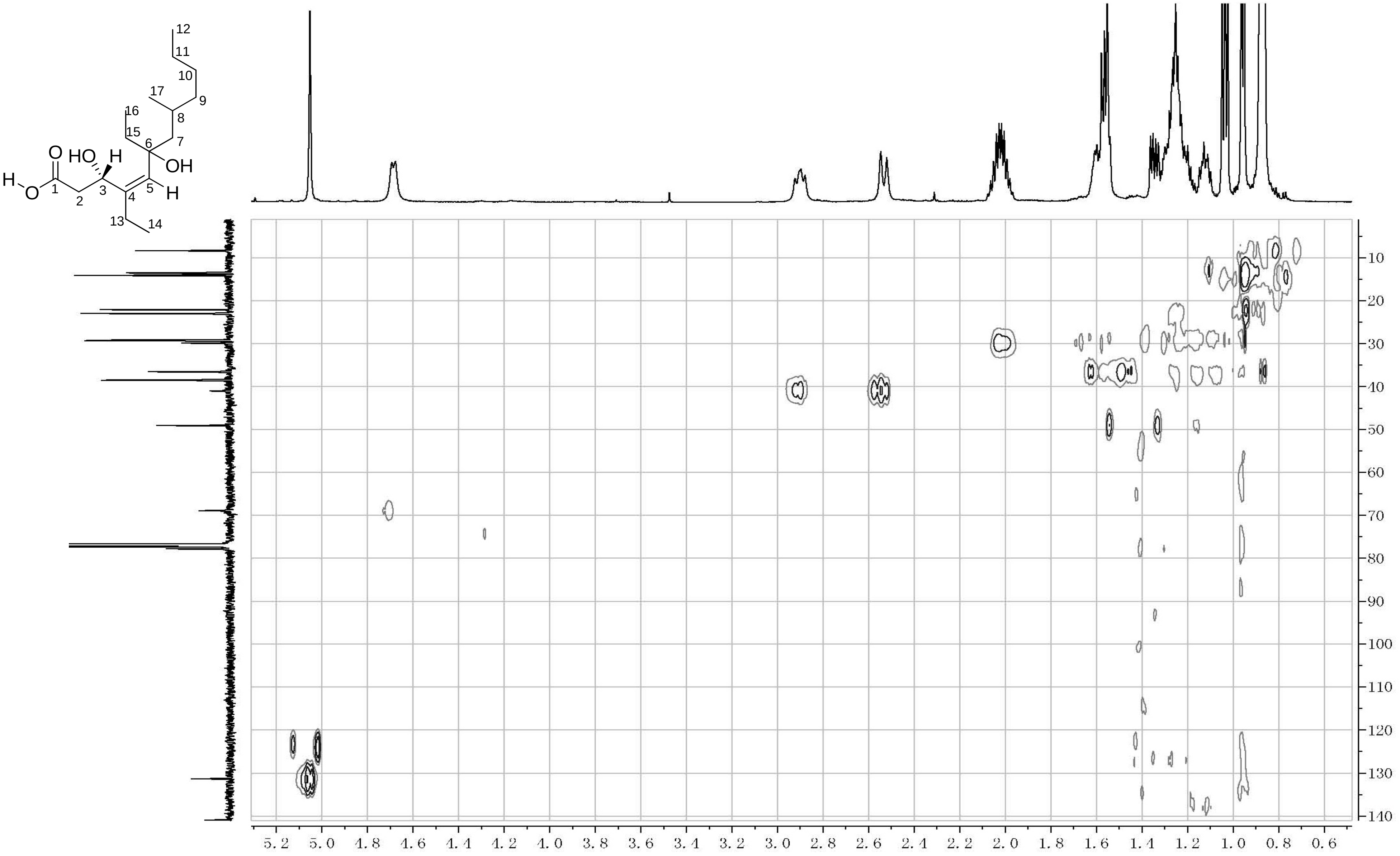

S23. HMBC spectrum of woodylide C (**3**) in $CDCl_3$.

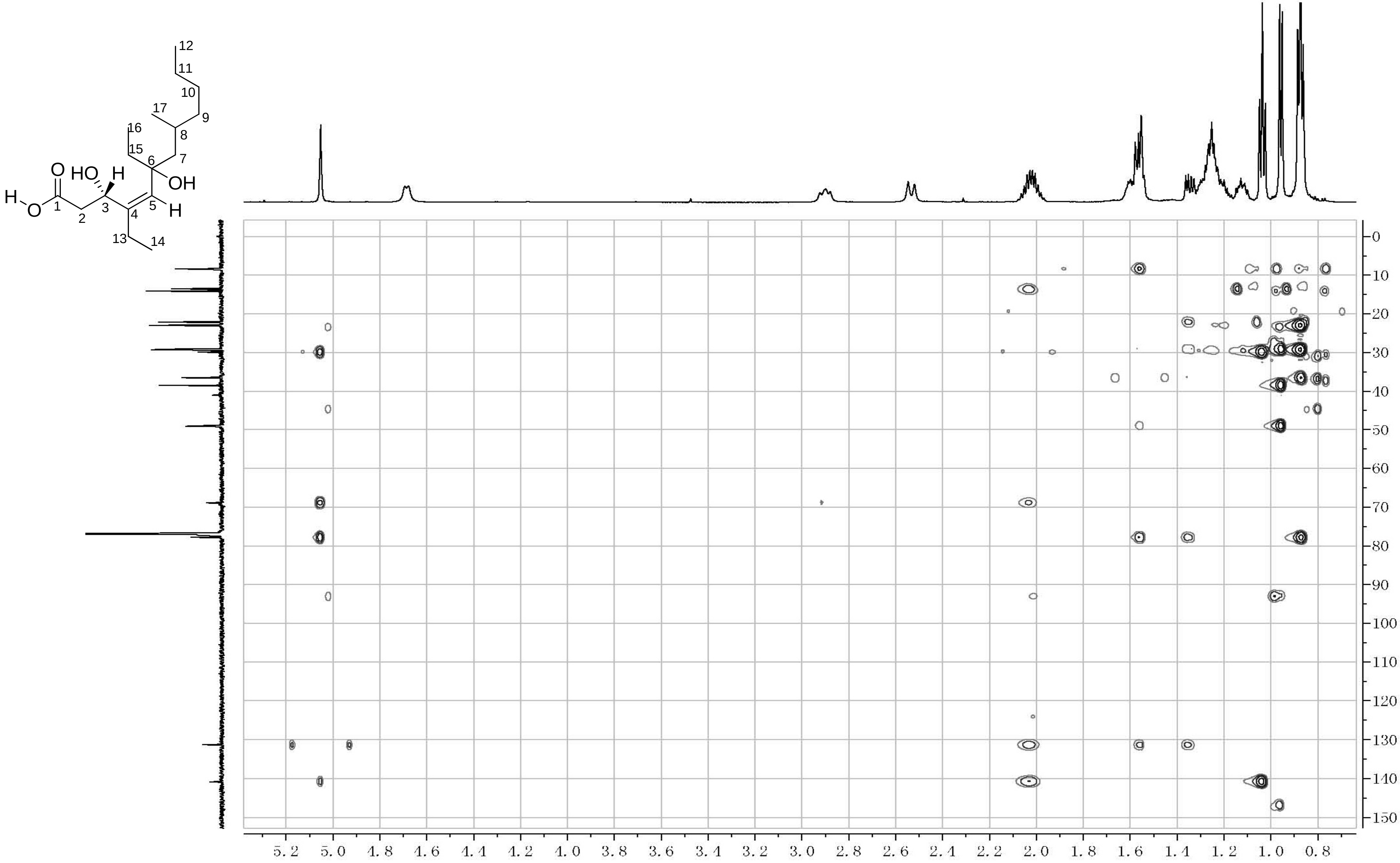

S24. ^{1}H-^{1}H COSY spectrum of woodylide C (**3**) in $CDCl_3$.

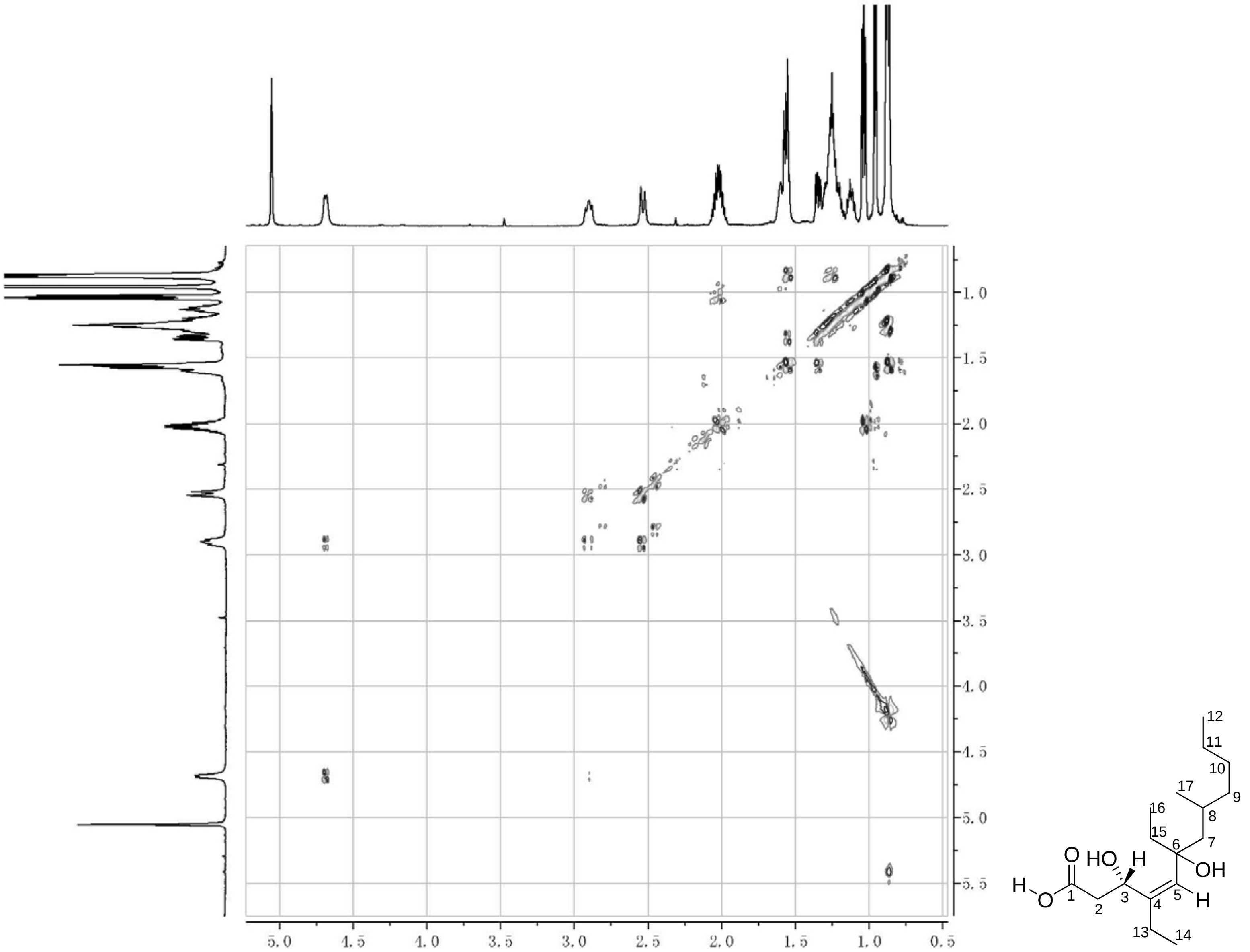

S25. NOESY spectrum of woodylide C (**3**) in $CDCl_3$.

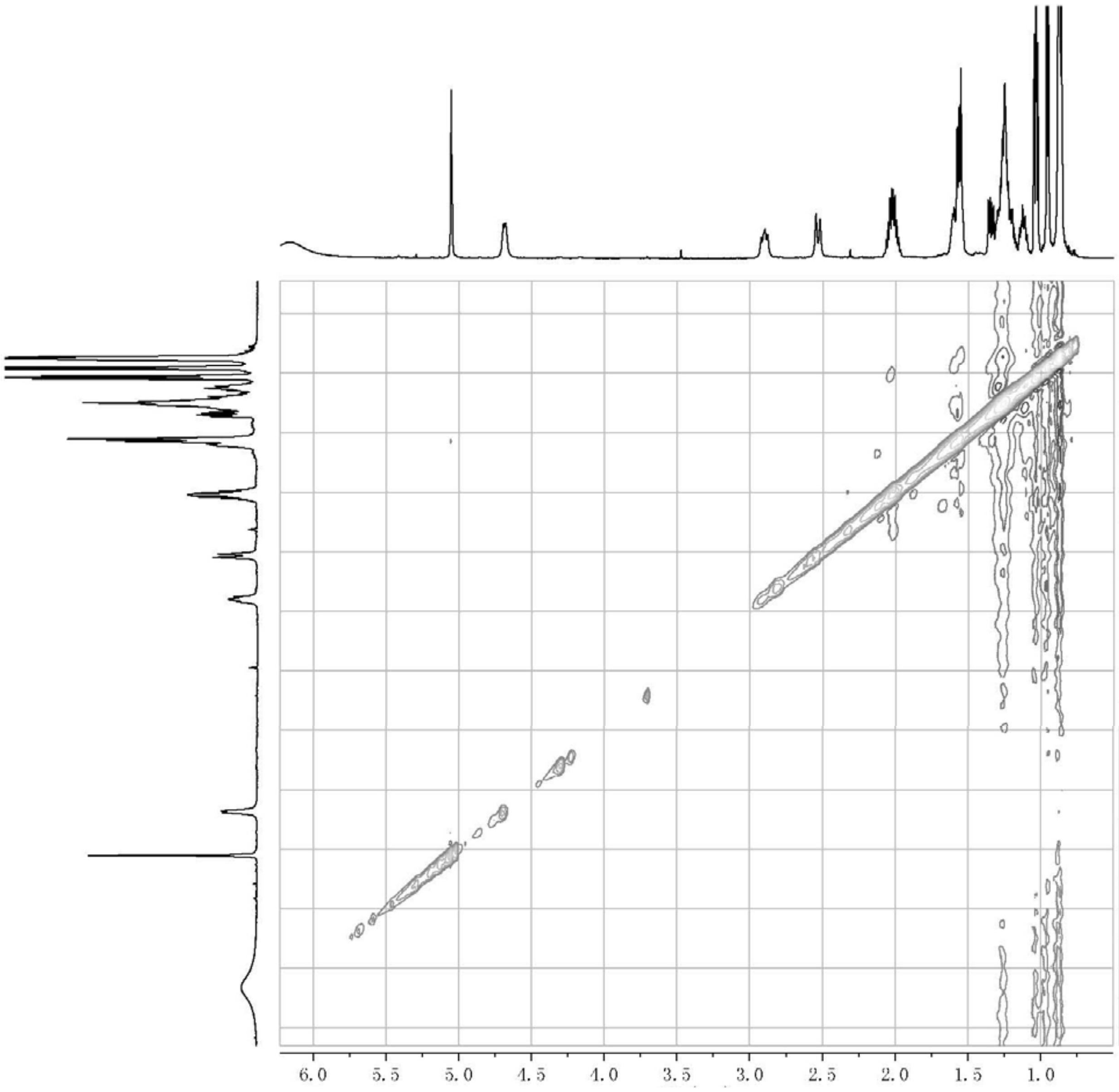

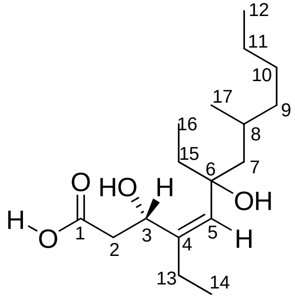

S26. IR spectrum of woodylide C (**3**).

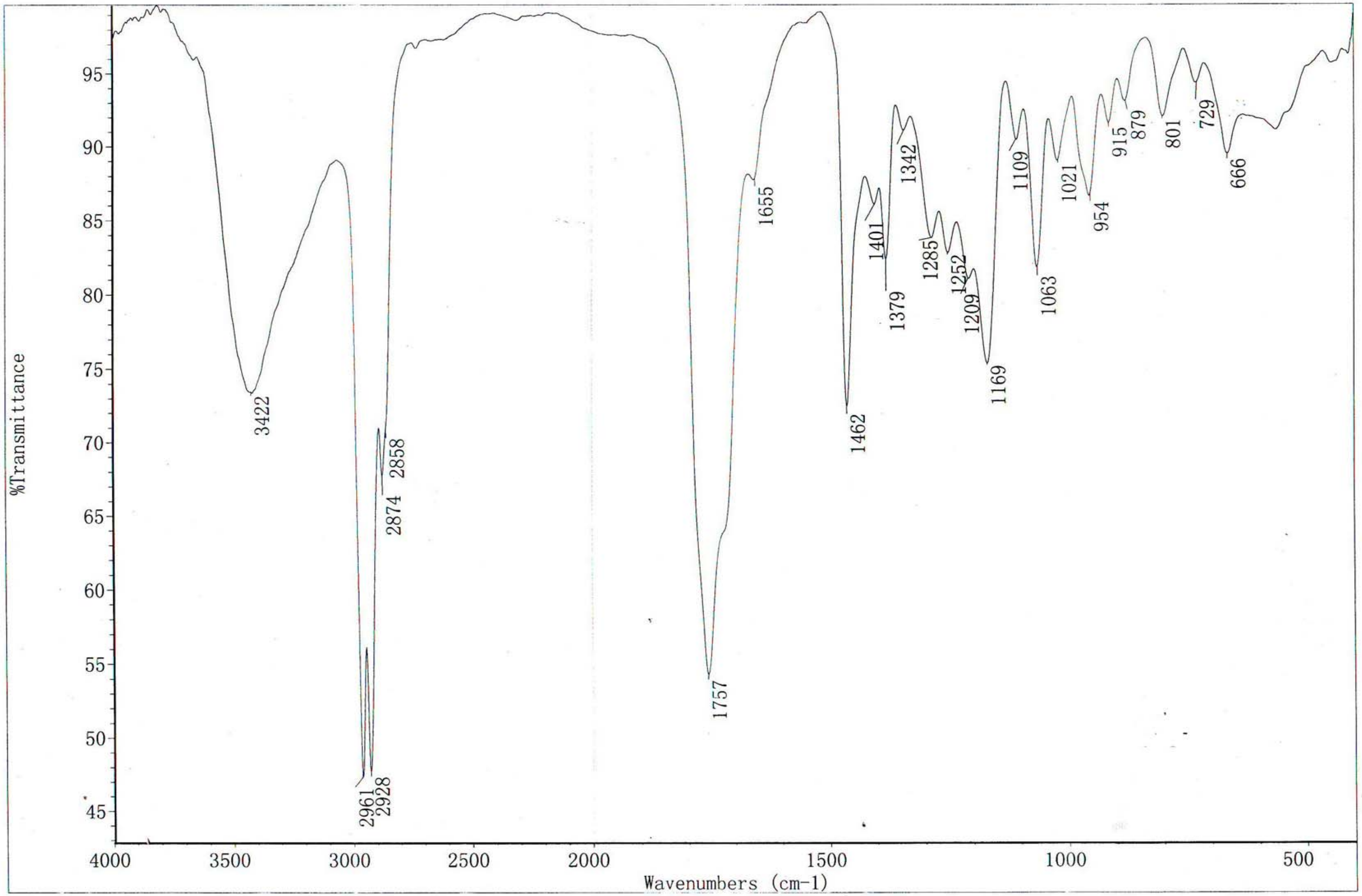

S27. HRESIMS of woodylide C (**3**).

Elemental Composition Report

Page 1

Tolerance = 10.0 PPM / DBE: min = -1.5, max = 50.0
Selected filters: None

Monoisotopic Mass, Even Electron Ions
4 formula(e) evaluated with 1 results within limits (up to 50 closest results for each mass)
Elements Used:
C: 5-17 H: 5-35 O: 1-4 Na: 1-1

SIPI

Q-Tof micro
YA019

02-Dec-2010,16:59:50
0.00000000
TOF MS ES+
3.09e4

WQ10594H 11 (0.380) AM (Cen,6, 80.00, Ar,5000.0,318.28,1.00); Sm (SG, 2x3.00); Cm (4:11)

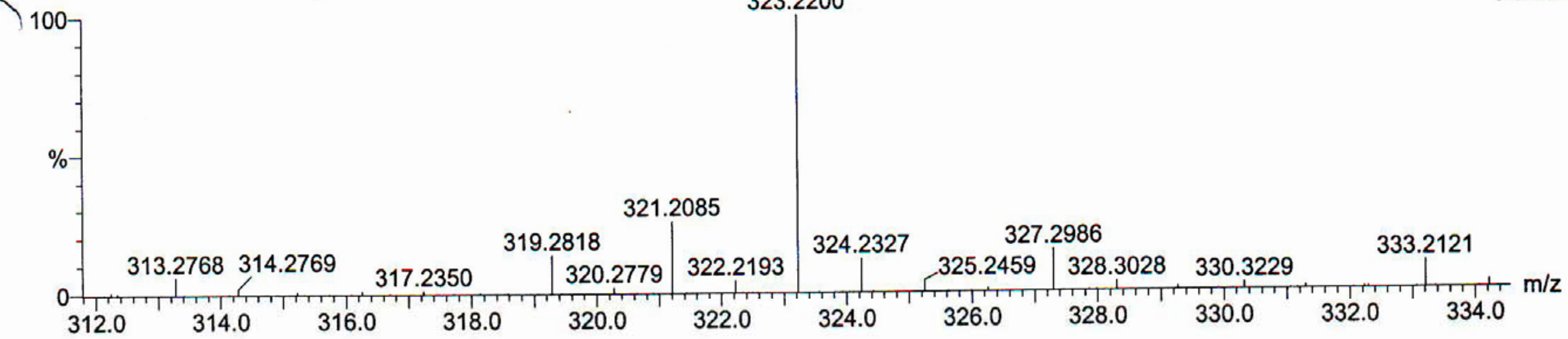

Minimum:	40.00				-1.5		
Maximum:	100.00		5.0	10.0	50.0		
Mass	RA	Calc. Mass	mDa	PPM	DBE	i-FIT	Formula
323.2200	100.00	323.2198	0.2	0.6	1.5	664.7	C17 H32 O4 Na

S28. CD spectrum of woodylides A–C (**1**–**3**).

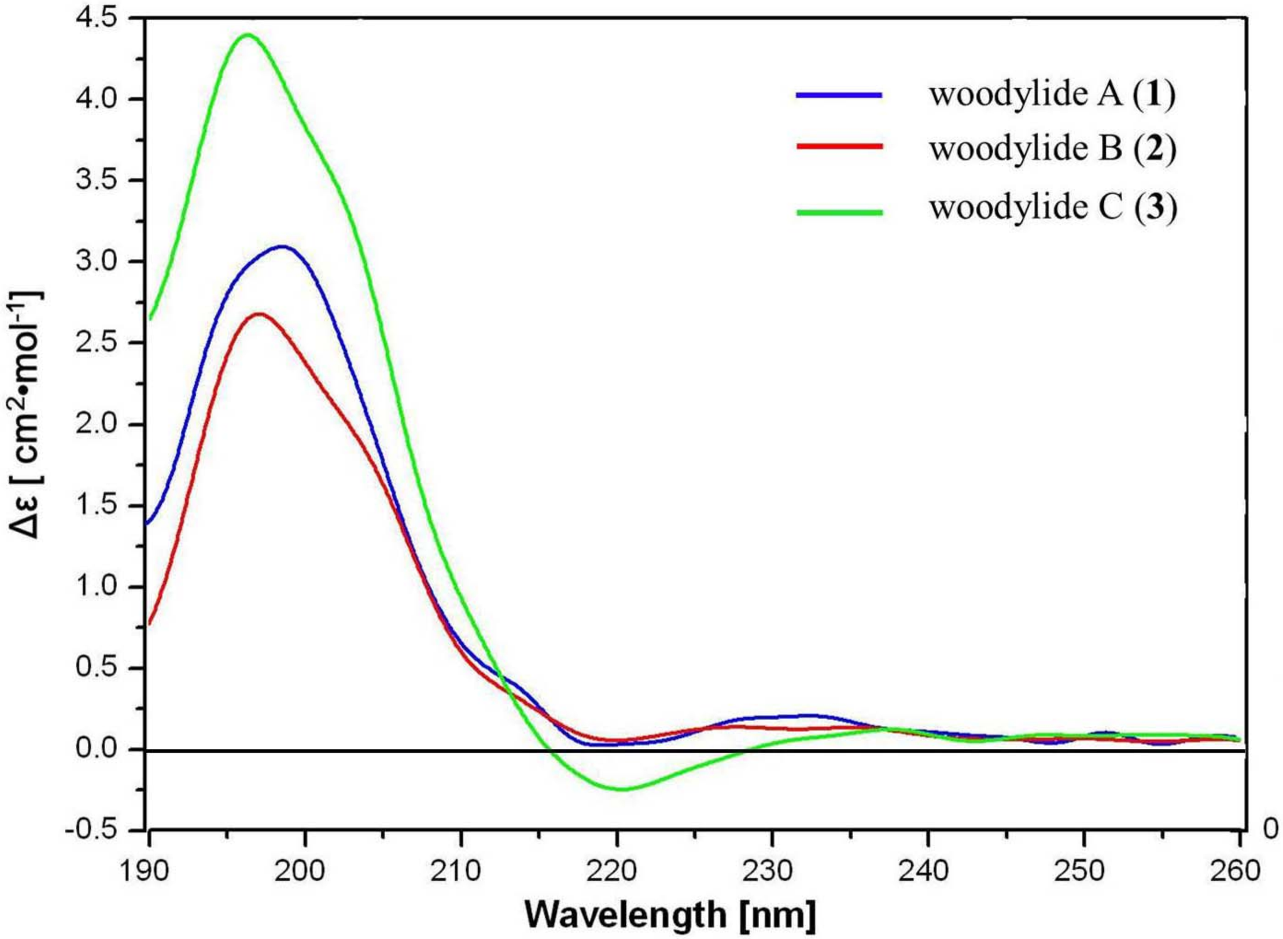

Supplement: Supplementary File 1: — PDF-Document (PDF, 3766 KB) [file marinedrugs-10-01027-s001.pdf]
